# First-principles investigation and photovoltaic assessment of Cs_2_SnZ_6_ (Z = Cl, Br, I) lead-free perovskites for future solar technologies

**DOI:** 10.1039/d5ra03487f

**Published:** 2025-07-24

**Authors:** Md. Ferdous Rahman, Abu Saiyed, Md. Faruk Hossain, Latha Marasamy, Tanvir Al Galib, Mahabur Rahman, Mutasem Z. Bani-Fwaz

**Affiliations:** a Advanced Energy Materials and Solar Cell Research Laboratory, Department of Electrical and Electronic Engineering, Begum Rokeya University Rangpur 5400 Bangladesh ferdousapee@gmail.com; b Department of Physics, Rajshahi University of Engineering & Technology Rajshahi 6204 Bangladesh; c Facultad de Química, Materiales-Energía, Universidad Autónoma de Querétaro Santiago de Querétaro Querétaro C.P.76010 Mexico; d Department of Chemistry, College of Science, King Khalid University P. O. Box 9004 Abha 61413 Saudi Arabia

## Abstract

This study presents a comprehensive first-principles and device-level investigation into the structural, electronic, optical, and photovoltaic properties of vacancy-ordered lead-free perovskites Cs_2_SnZ_6_ (Z = Cl, Br, I) for next-generation solar energy conversion. Using density functional theory (DFT), we examined the structural stability, band structure, density of states, and optical response of Cs_2_SnCl_6_, Cs_2_SnBr_6_, and Cs_2_SnI_6_. The calculated direct bandgaps are 2.652 eV for Cs_2_SnCl_6_, 1.358 eV for Cs_2_SnBr_6_, and 0.228 eV for Cs_2_SnI_6_, demonstrating significant tunability through halide substitution. Optical analyses reveal strong absorption in the visible spectrum, with a redshift in absorption onset from Cl to I, enhancing light-harvesting capabilities. To assess device performance, SCAPS-1D simulations were employed with four different electron transport layers (ETLs): CdS, IGZO, SnS_2_, and ZnS. The Cs_2_SnBr_6_-based PSC with IGZO ETL achieved the highest power conversion efficiency (PCE) of 26.22%, driven by optimal band alignment and balanced charge transport. Meanwhile, Cs_2_SnI_6_, despite exhibiting ultrahigh short-circuit current densities (*J*_SC_ > 70 mA cm^−2^) due to its narrow bandgap, showed poor performance with ZnS ETL due to mismatched energy levels. These results highlight the potential of Cs_2_SnZ_6_ perovskites as promising lead-free absorber materials and emphasize the critical role of ETL compatibility and absorber optimization in achieving high-efficiency solar cells.

## Abstract 2

This study explores the structural, electronic, optical, and photovoltaic properties of lead-free vacancy-ordered perovskites Cs_2_SnZ_6_ (Z = Cl, Br, I) *via* first-principles and device-level methods for next-generation solar energy applications.

## Graphical abstract

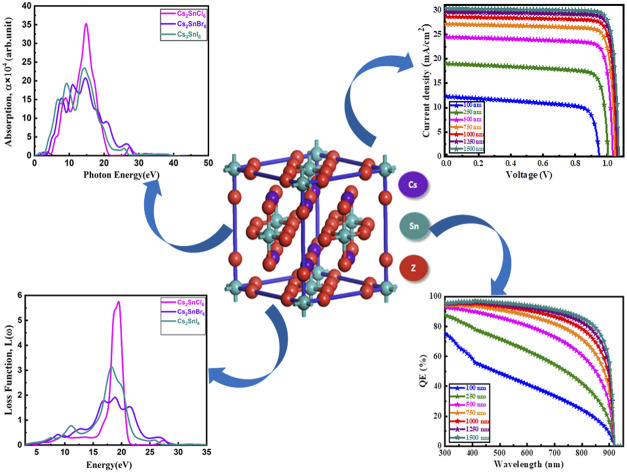

## 1. Introduction

The quest for clean, renewable, and sustainable energy sources has become an urgent global imperative amidst rising environmental concerns, climate change, and the pressing need to decarbonize the global energy economy.^1,2^ Among the diverse range of renewable energy systems, photovoltaic (PV) solar energy stands at the forefront due to its abundance, scalability, and minimal environmental impact. Silicon (Si) has emerged as the predominant material for commercial solar cell applications owing to its favorable properties, including stability, high power conversion efficiency (PCE), and resistance to radiation.^3^ The development of conventional solar cells, particularly those based on silicon (Si), has been limited by inherent drawbacks, including silicon's indirect and suboptimal bandgap, as well as the high costs associated with its fabrication processes.^4^ In recent decades, halide perovskite solar cells (PSCs) have garnered extraordinary interest owing to their rapid rise in PCE, reaching over 26% within a decade, along with their low-cost processing and tunable optoelectronic properties.^5–13^ However, the commercialization of perovskite-based PV devices remains hindered by issues of toxicity, primarily due to the inclusion of lead (Pb), and long-term material instability under operational conditions.^14,15^ This has stimulated intensive research into the discovery and optimization of lead-free perovskite alternatives that combine environmental benignity with high photovoltaic performance. Although extensive efforts have been devoted to developing lead-free perovskite materials, attaining PCE and environmental stability on par with their lead-based counterparts remains a significant challenge.^16,17^ Therefore, there is a pressing demand within the photovoltaic (PV) sector to explore lead-free, high-efficiency materials that are viable for large-scale commercial deployment.^18^

A promising class of lead-free perovskites under exploration is based on vacancy-ordered double perovskite structures of the form A_2_BIVZ_6_, where A is a monovalent cation (*e.g.*, Cs^+^), BIV is a group-IV metal such as Sn^4+^, and Z is a halide anion (Cl^−^, Br^−^, I^−^).^19^ Among these, cesium tin halide perovskites, Cs_2_SnZ_6_ (Z = Cl, Br, I), have emerged as compelling candidates due to their favorable structural stability, moderate direct bandgaps, and nontoxic nature.^20^ Moreover, Cs_2_SnZ_6_ exhibits a high-symmetry arrangement in the *Fm*3̄*m* space group, similar to the structure of cubic halide perovskites.^21–23^ Notably, the vacancy-ordered Cs_2_SnZ_6_ compounds differ fundamentally from their Sn^2+^-based counterparts (*e.g.*, CsSnZ_3_), offering enhanced resistance to oxidation and improved ambient stability, which is a pivotal factor in ensuring long-term device performance.^24,25^ Despite these advantages, their practical photovoltaic potential remains underexplored, particularly in relation to their interfacial alignment with charge transport layers, which plays a crucial role in governing charge separation and extraction dynamics in solar cell architectures.

Recent simulation-based investigations on lead-free double perovskites such as Cs_2_AgBiBr_6_ and Cs_2_SnI_6_ have demonstrated promising optoelectronic performance and environmental stability, making them suitable candidates for photovoltaic applications.^26–28^ For instance, Cs_2_AgBiBr_6_ has shown respectable PCE values in numerical models under optimized conditions,^26^ while Cs_2_SnI_6_ exhibits favorable bandgap alignment and defect tolerance as validated by SCAPS-1D and DFT studies.^28^ These studies highlight the potential of vacancy-ordered and mixed-halide perovskites, motivating the exploration of alternative Sn-based compositions such as Cs_2_SnZ_6_.

First-principles density functional theory (FP-DFT) calculations offer a robust platform for probing the intrinsic material properties of perovskites at the atomic scale, enabling insights into electronic band structure, optical absorption characteristics, effective masses, and defect tolerance. When complemented with device-level simulations, such as those conducted using SCAPS-1D software, a comprehensive evaluation of the photovoltaic behavior of these materials can be achieved. This dual-level approach allows for the rational selection of compatible electron transport layers (ETLs) that are critical for optimizing band alignment, minimizing recombination losses, and enhancing overall device efficiency. In this context, CdS, IGZO (indium gallium zinc oxide), SnS_2_, and ZnS have been identified as promising ETLs due to their appropriate bandgaps, high electron mobility, and chemical compatibility with various perovskites.^29–31^

In this study, we present a comprehensive theoretical and numerical investigation into the structural, electronic, and optical properties of Cs_2_SnZ_6_ compounds using first-principles density functional theory. We systematically evaluate their suitability as absorber materials in lead-free PSCs, focusing on bandgap tunability, absorption coefficients, dielectric response, and charge transport properties. Subsequently, we assess the photovoltaic performance of Cs_2_SnZ_6_-based solar cells using SCAPS-1D simulations, incorporating four different ETLs: CdS, IGZO, SnS_2_, and ZnS. Through detailed energy band alignment analysis and performance metrics including open-circuit voltage (*V*_OC_), short-circuit current density (*J*_SC_), fill factor (FF), and overall PCE, we identify the optimal material configurations for enhanced device performance. This integrative approach provides valuable insights into the interfacial and device-level phenomena that govern the operation of lead-free perovskite solar cells, offering a strategic pathway for the development of stable, efficient, and environmentally benign photovoltaic technologies.

Our findings underscore the immense potential of Cs_2_SnZ_6_ halides as next-generation photovoltaic absorbers and demonstrate the critical influence of ETL selection on the efficiency and stability of the resulting devices. The implications of this work extend beyond material discovery to encompass device engineering strategies that can accelerate the deployment of lead-free perovskite photovoltaics in real-world applications.

## 2. Simulation methodology with DFT and SCAPS-1D

The structural and electronic properties of Cs_2_SnZ_6_ compounds were thoroughly investigated using an integrated computational methodology that combines advanced theoretical tools. Specifically, first-principles density functional theory (DFT) calculations^32^ were carried out to gain deep insights into the behavior of these materials at the atomic level. The simulations employed on-the-fly generated (OTFG) ultrasoft pseudopotentials,^33,34^ which are well-suited for achieving high accuracy in modeling complex materials systems. Additionally, the Perdew–Burke–Ernzerhof (PBE) exchange–correlation functional, within the generalized gradient approximation (GGA) framework,^35^ was used to describe electron interactions. This approach enabled precise evaluation of the ground-state electronic structure, bonding nature, and stability of Cs_2_SnZ_6_ compounds, leveraging the robust predictive power of DFT.^33^

The computational protocol incorporated essential input parameters such as lattice constants, Brillouin zone sampling grids, crystal structure data, and kinetic energy cut-off values. These parameters were judiciously selected to ensure reliable and precise simulation outcomes within the DFT framework.^18,36,37^ To enhance the accuracy of structural optimizations and electronic property evaluations, a kinetic energy cut-off of 750 eV was employed for all Cs_2_SnZ_6_ configurations.^38^

For self-consistent field (SCF) calculations, the energy convergence criterion was set to 10^−6^ atomic units, with the highest force threshold of 0.01 eV Å^−1^ is ensured accurate ground-state energy determinations. During structural relaxation and ionic optimization, a force convergence limit of 10^−4^ atomic units was applied. Band structure and partial density of states (PDOS) calculations utilized a Monkhorst–Pack *k*-point mesh of 10 × 10 × 10 to achieve high-resolution sampling of the Brillouin zone.^39^

The optical properties of the materials were investigated through first-order time-dependent perturbation theory, which enabled the analysis of dynamic structural stability and optoelectronic response. In particular, the frequency-dependent complex dielectric function, *ε*(*ω*) = *ε*_1_(*ω*) + *jε*_2_(*ω*), was computed to elucidate the interaction between incident photons and the material system. This function facilitated the determination of optical absorption coefficients across a broad photon energy spectrum (in electron volts, eV), providing insight into light–matter interactions and energy harvesting potential. For these calculations, a Gamma-centered 10 × 10 × 10 Monkhorst–Pack *k*-mesh was adopted to maintain consistency with structural and electronic evaluations.

To simulate device-level performance, the one-dimensional Solar Cell Capacitance Simulator (SCAPS-1D), developed by the Department of Electronics and Information Systems (ELIS) at Ghent University, Belgium, was employed. The simulated photovoltaic device structure comprised Al/FTO/ETL/Cs_2_SnZ_6_/Au layers. Through this framework, the electrical behavior of the heterojunction was modeled by solving the coupled continuity equations for electrons and holes, as well as the Poisson equation under equilibrium conditions. The simulations rigorously assessed carrier transport dynamics and energy band alignment, enabling a comprehensive evaluation of the photovoltaic potential of Cs_2_SnZ_6_-based lead-free perovskite solar cells.^40,41^

## 3. Result and discussion

### 3.1 DFT-based investigation of new inorganic cubic perovskites Cs_2_SnZ_6_

#### 3.1.1 Structural properties

**Fig. 1:**
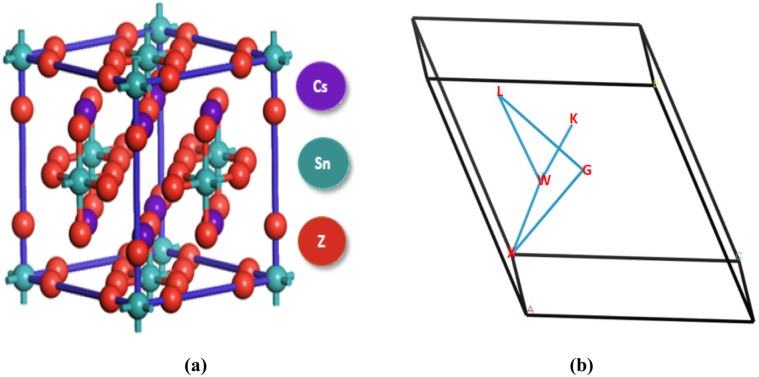
(a) Crystal structure of Cs_2_SnZ_6_ compound, and (b) the initial Brillouin zone (BZ) *k*-vector trajectory.

Cs_2_SnZ_6_ is a well-defined inorganic halide compound crystallizing in the cubic *Fm*3̄*m* space group with space group number 225.^19,42,43^ Structurally, it comprises cesium (Cs) as the monovalent cation and a combination of tetravalent tin (Sn) and halide anions (Cl, Br, or I), forming a highly symmetric cubic lattice. The unit cell of Cs_2_SnZ_6_ consists of nine atoms, with Cs, Sn, and Z atoms occupying distinct Wyckoff positions. Specifically, the halide atom Z (Cl/Br/I) is located at fractional coordinates (0.25, 0.25, 0.75), while the four Sn atoms are symmetrically positioned at (0, 0, 0). The corresponding halide atoms, such as Br in the case of Cs_2_SnBr_6_, occupy positions at (0, 0.5, 0.75). This atomic arrangement is visually represented in Fig. 1(a), which depicts the precise crystallographic configuration.

Fig. 1(b) illustrates the high-symmetry path of the *k*-vector within the first Brillouin zone (BZ), which is essential for evaluating the electronic band structure. The calculated band structures of Cs_2_SnZ_6_ compounds are significantly governed by transitions near the high-symmetry points W, L, G, X, and W. These points critically determine the electronic dispersion and the overall shape of the band diagram. Using first-principles computational techniques, the optimized lattice constants were determined to be approximately 10.49 Å for Cs_2_SnCl_6_, 10.99 Å for Cs_2_SnBr_6_, and 11.78 Å for Cs_2_SnI_6_, indicating a systematic lattice expansion with increasing halide ionic radius.

#### 3.1.2 Electronic properties

**Fig. 2:**
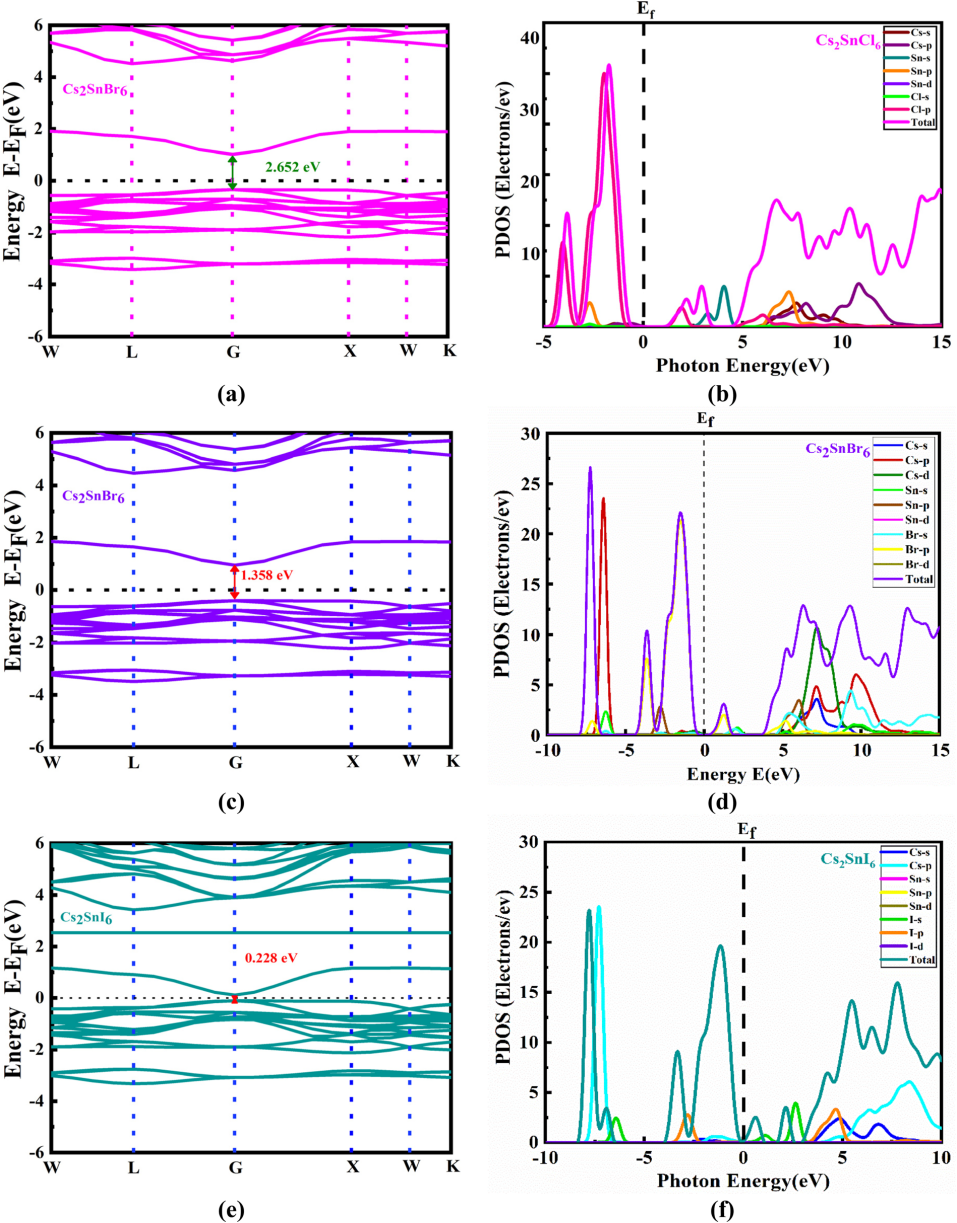
(a) Electronic band structure and (b) PDOS of Cs_2_SnCl_6_, (c) electronic band structure and (d) PDOS of Cs_2_SnBr_6_, and (e) electronic band structure and (f) PDOS of Cs_2_SnI_6_.

The electronic band structures of the inorganic cubic substance Cs_2_SnZ_6_ were computed to improve our comprehension of its optical and electronic properties. Fig. 2(a–c) illustrates the electronic band structures of the Cs_2_SnZ_6_ framework. Fig. 2 presents the electronic band structures of Cs_2_SnZ_6_, with the Fermi level aligned at zero to facilitate accurate determination of the bandgap. The reciprocal space trajectory traced along the high-symmetry points W–L–G–X–W is employed to characterize the band dispersion within the cubic Brillouin zone. For all three compounds—Cs_2_SnCl_6_, Cs_2_SnBr_6_, and Cs_2_SnI_6_—both the valence band maximum (VBM) and conduction band minimum (CBM) are located at the Γ point, indicating a direct bandgap nature, as clearly illustrated in Fig. 2. The G point represents the center of the Brillouin zone, while W, L, and X denote critical high-symmetry points that define the key electronic transitions in the crystal momentum space.

The calculated direct bandgaps using the generalized gradient approximation (GGA-PBE) exchange–correlation functional are 2.652 eV for Cs_2_SnCl_6_, 1.358 eV for Cs_2_SnBr_6_, and 0.228 eV for Cs_2_SnI_6_. These values reveal a clear trend of bandgap narrowing with increasing halide ionic radius, which can be attributed to the decreasing electronegativity and enhanced orbital overlap in larger halide anions. As the electronegativity of the halide decreases from Cl to I, the bonding nature becomes increasingly ionic to covalent, which enhances the overlap between the halide p-orbitals and the Sn-5p orbitals. This increased orbital overlap results in stronger antibonding interactions at the VBM and CBM, leading to a reduction in the bandgap energy.^23,44^ Additionally, larger halide ions (like I^−^) cause lattice expansion, which reduces the crystal field splitting and further narrows the bandgap.^23^ This trend is explicitly presented in Table 1, highlighting the influence of halide substitution on electronic structure. The relatively wide bandgap of Cs_2_SnCl_6_ contrasts with the narrower gaps of Cs_2_SnBr_6_ and Cs_2_SnI_6_, underscoring the tunability of these materials for specific optoelectronic applications. Notably, the presence of a direct bandgap makes these compounds particularly suitable for photovoltaic and optoelectronic devices, where efficient photon absorption and electron–hole generation are essential.^44–46^ The distinct band gaps and Fermi level positioned near the valence band (VB), along with a higher density of states in the VB than in the conduction band (CB), confirm the p-type semiconducting nature of these materials.^47^

**Table 1:** The variation of lattice constant, unit-cell volume, and direct energy bandgap of Cs_2_SnZ_6_ in GGA-PBE function

Material	Lattice constant (Å)	Bandgap (eV)	Unit-cell volume (a.u.)^3^
Cs_2_SnCl_6_	10.49	2.652 (GGA-PBE)	1155.34
Cs_2_SnBr_6_	10.99	1.358 (GGA-PBE)	1326.03
Cs_2_SnI_6_	11.78	0.228 (GGA-PBE)	1634.48

To elucidate the influence of various atomic species and their corresponding electronic states on the bandgap characteristics of Cs_2_SnZ_6_ compounds, we performed detailed Partial Density of States (PDOS) calculations. This analysis was aimed at achieving a deeper understanding of how specific atomic orbitals contribute to the formation and modulation of the electronic bandgap. As illustrated in Fig. 2(b), (d) and (f), the PDOS profiles span the energy range from −10 eV to +10 eV, highlighting the energy distribution and orbital contributions of Cs, Sn, and halide atoms to the electronic structure of the compounds. The PDOS results reveal significant orbital hybridization between the electronic states of cesium (Cs) and the halide ions (Cl^−^, Br^−^, I^−^) with tin (Sn), which is sustained across a wide energy interval. This hybridization coexists with the preservation of a finite bandgap, confirming the semiconducting nature of these compounds. A prominent observation is the weak involvement of Cs electronic states near the Fermi level; Cs orbitals reside far from the conduction and VB edges, and therefore, their direct contribution to the band-edge states is minimal. Additionally, electron density transfer from Cs atoms to Sn and halide atoms suggests notable charge redistribution within the lattice.

For all Cs_2_SnZ_6_ compounds, the VBM is primarily governed by antibonding interactions involving Sn-5p and halogen-p orbitals, while the CBM arises mainly from Sn-5p and Cs-5d antibonding orbitals, as depicted in Fig. 2. In the valence region, the Br-p orbitals dominate across all compounds, while additional contributions are observed from Cs-p and Cl-s orbitals in Cs_2_SnCl_6_, Cs-p, Sn-d, and Br-d orbitals in Cs_2_SnBr_6_, and Cs-p, Sn-d, and I-d orbitals in Cs_2_SnI_6_, respectively. The substitution of halide anions introduces notable variations in both the PDOS and the Total Density of States (TDOS), as reflected in the PDOS curves. Distinct shifts in the peak intensities of the TDOS are observed across the three compounds, with maximum values measured at 35.85 for Cs_2_SnCl_6_, 26.60 for Cs_2_SnBr_6_, and 23.42 for Cs_2_SnI_6_, respectively. These disparities underscore the pronounced impact of halide substitution on the electronic structure, altering the density and distribution of states across the energy spectrum. The observed modifications in the TDOS highlight the unique electronic fingerprints of each compound and affirm the role of Z-anion engineering in tuning the optoelectronic properties of Cs_2_SnZ_6_ perovskites for photovoltaic applications.

#### 3.1.3 Optical properties

Analyzing the optical properties of materials is a fundamental step in determining their suitability for optoelectronic and photovoltaic applications. Such analyses typically involve evaluating key optical parameters, including the real and imaginary parts of the dielectric function, optical absorption spectrum, and the electron energy loss function (ELF). The frequency-dependent complex dielectric function, represented as *ε*(*ω*), characterizes a material's response to incident electromagnetic radiation and reflects its complex permittivity. This function is composed of two primary components: the real part, *ε*_1_(*ω*), which corresponds to the dispersion of the electromagnetic wave, and the imaginary part, *ε*_2_(*ω*), which represents the absorption of energy from the field. These relationships are formally expressed as:

1 *ε*(*ω*) = *ε*_1_(*ω*) + *iε*_2_(*ω*)

The *ε*_1_(*ω*) is computed through the application of the Kramers–Kronig transformation, a fundamental mathematical technique that derives *ε*_1_(*ω*) from the frequency-dependent behavior of the *ε*_2_(*ω*).^47–52^ This transformation establishes a causal relationship between the two components of the complex dielectric function. In contrast, the *ε*_2_(*ω*) is determined based on the momentum matrix elements, which quantify electronic transitions between occupied and unoccupied states under photon excitation.^49,50,54,55^ Furthermore, essential optical parameters such as the absorption coefficient (*α*) and the electron energy loss function (ELF) can be directly extracted from the real and imaginary parts of the dielectric function.^53^ These properties are pivotal in assessing a material's interaction with electromagnetic radiation and its potential efficacy in optoelectronic applications.^48–50,56^

The expression for the real component of the dielectric function, denoted as *ε*_1_(*ω*), can be formulated as follows:^56,57^2
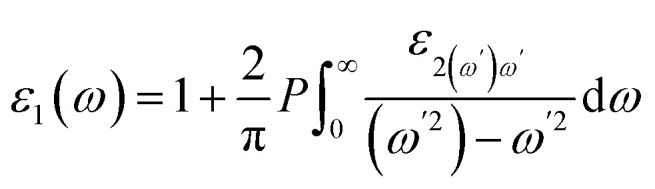
Here, *P* represents the integral prime value.

The mathematical expression for the *ε*_2_(*ω*), in terms of the momentum operator, can be formally represented as follows:^56,57^3
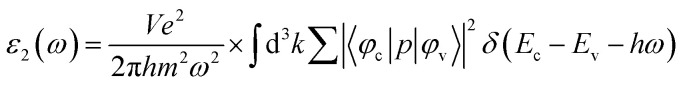
In this formulation, *V* denotes the volume of the unit cell, ℏ represents the reduced Planck constant, and *p* corresponds to the momentum operator. The symbols *ϕ*_c_ and *ϕ*_v_ refer to the wave functions of the VB and CB, respectively, while *E*_c_ and *E*_v_ indicate the energy levels of the CB and VB. Additionally, *δ* represents the Dirac delta function, which enforces energy conservation during electronic transitions. Utilizing both the real component *ε*_1_(*ω*) and the imaginary component *ε*_2_(*ω*) of the complex dielectric function, the optical absorption coefficient *α* can be evaluated using the following relation:^56,57^:4
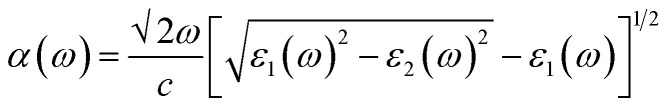
In this expression, *ω* denotes the angular frequency of the incident electromagnetic radiation, while *c* refers to the speed of light in a vacuum. Finally, the electron energy loss function (ELF), which characterizes the energy dissipated by fast electrons traversing the material, can be computed using the following relation:^48,56^5
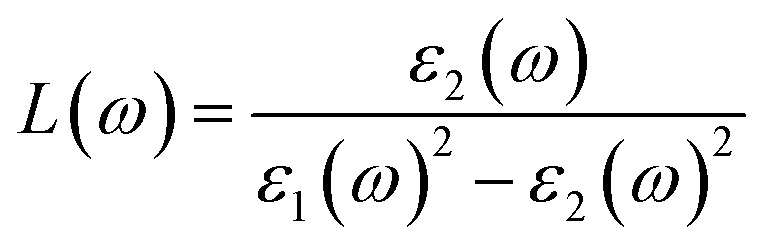


##### 3.1.3.1 Real function

**Fig. 3:**
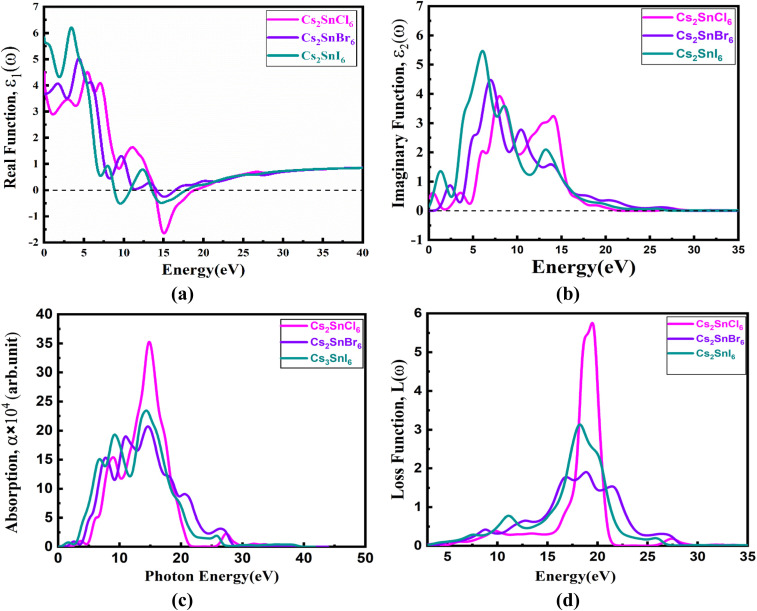
The energy-dependent (a) dielectric real function, (b) dielectric imaginary function, (c) absorption index, and (d) loss function.

Fig. 3(a) presents the real part of the dielectric function, *ε*_1_(*ω*), for Cs_2_SnZ_6_ compounds across a photon energy range extending up to 7 eV. The real component, *ε*_1_(*ω*), reflects the material's polarizability and dispersive behavior in response to an externally applied electric field, offering critical insight into its dielectric screening and refractive characteristics.^52,58^ Each compound shows a positive *ε*_1_(*ω*) within the 0–5 eV energy range, which is characteristic of semiconducting materials.^59^ The parameter *ε*_1_(0), representing the static dielectric constant or the electronic contribution at zero frequency, plays a pivotal role in characterizing a material's intrinsic optical response and is directly obtained from the real part of the dielectric function, *ε*_1_(*ω*). For the cubic perovskite compounds Cs_2_SnCl_6_, Cs_2_SnBr_6_, and Cs_2_SnI_6_, the calculated *ε*_1_(0) values are 4.51, 3.66, and 5.83, respectively. The lowest *ε*_1_(0) observed for K_2_InSbBr_6_ among the halide series can be attributed to the bandgap–dielectric relationship described by the Penn model, where reduced electronic polarizability leads to lower dielectric response in materials with wider bandgaps.^60^ Although K_2_InSbBr_6_ does not exhibit the largest bandgap, its lower density of electronic states near the band edges and less effective orbital overlap—compared to the F and I analogs—result in diminished dielectric screening and reduced *ε*_1_(0). Distinctive trends are observed in the spectral behavior of *ε*_1_(*ω*) when these materials are subjected to incident optical radiation. The real dielectric component initially increases from its static value, reaching a peak at specific photon energies, and subsequently undergoes a sharp decline. This characteristic response indicates a strong dielectric polarization within a limited energy range, reflecting the material's capability to interact efficiently with electromagnetic radiation.

Generally, materials with larger bandgaps tend to exhibit lower peak values in their dielectric functions due to reduced electronic polarizability, whereas those with narrower bandgaps show stronger optical responses. As the halide anion in Cs_2_SnZ_6_ is varied from Cl to I, a noticeable blueshift in the dielectric function is observed, wherein the *ε*_1_(*ω*) peak shifts toward higher photon energies. This shift is attributed to the increase in bandgap energy, which modifies the onset of interband transitions and alters the material's optical dispersion characteristics. Conversely, as the halogen size increases, the peak intensity rises, likely due to the enhanced polarizability associated with bigger halogen atoms.^61^

##### 3.1.3.2 Imaginary function

Fig. 3(b) depicts the spectral behavior of the *ε*_2_(*ω*), for Cs_2_SnZ_6_ compounds with halide substitutions. The *ε*_2_(*ω*) provides critical insights into the electronic bandgap characteristics and the material's optical absorption properties, as it directly reflects the interband electronic transitions induced by incident photons.^5^ It is intricately associated with the material's band structure in the previous studies.^58^ The values of *ε*_2_(*ω*)for Cs_2_SnZ_6_ materials exhibit a wide absorption spectrum, reflecting their diverse absorption capabilities. As shown in Fig. 3(b), the *ε*_2_(*ω*) peaks for Cs_2_SnCl_6_, Cs_2_SnBr_6_, and Cs_2_SnI_6_ are observed at optical energies of 3.92, 4.47, and 5.46 eV, corresponding to photon absorption energies of 7.98 eV, 6.98 eV, and 6.07 eV, respectively. These peaks in the imaginary component of the dielectric constant align with the transitions of carriers from the VB to the CB. The peak positions vary due to changes in the composition of the Z-anions. Notably, the spectra reveal a consistent trend where the peaks shift toward lower values as the size of the Z-anions increases from Cl to I in the Cs_2_SnZ_6_ structures. Furthermore, the materials under study exhibit an imaginary dielectric constant approaching zero at photon energies above 35 eV, indicating excellent optical transparency and minimal light absorption.

##### 3.1.3.3 Absorption coefficients

The optical absorption coefficient is crucial for understanding a material's ability to absorb and convert light energy into other forms, such as heat or electrical energy, making it essential for solar cell applications.^58,62^Fig. 3(c) illustrates the relationship between the absorption coefficient and photon energy for Cs_2_SnZ_6_ structures. In the context of solar cell applications, the primary absorption peak is particularly significant, as it indicates the wavelength range where a material can efficiently absorb electromagnetic radiation. The initial peak determines whether the Cs_2_SnZ_6_ structures can absorb within the visible light spectrum. Among the optical absorption spectra of all the materials, the most prominent peak is observed in the visible region.

The observed changes in absorption within Cs_2_SnZ_6_ closely correspond to the expected bandgap of the materials. In the visible spectrum, the absorption coefficient for Cs_2_SnZ_6_ increases as the size of the Z-anions grows, which is a desirable characteristic for solar cell applications. As the Z-anions transition from Cl to I, the material's bandgap decreases, resulting in a redshift of the absorption edge toward the lower energy region. Of the four materials, Cs_2_SnCl_6_ exhibits the highest absorption coefficient at 14.78 eV.

##### 3.1.3.4 Energy loss function (ELF)

The ELF quantifies the energy dissipation of electrons as they travel through a dielectric medium. The *L*(*ω*) representation of the ELF is valuable for examining a material's response to light. Fig. 3(d) shows that the ELF peaks for Cs_2_SnZ_6_, with a cubic structure, occur within the energy range of 15 to 22 eV. The relatively low magnitude of *L*(*ω*) in the visible region for the studied materias indicates that these halide compounds possess strong light absorption capabilities.^63^ The peaks in the ELF diagrams of Cs_2_SnZ_6_ correspond to energy dissipation that takes place when the energy of the incident photon exceeds the material's bandgap. It is clear that when the photon energy does not surpass the bandgap, no significant scattering occurs. Prominent peaks were observed at different energy levels for the Cs_2_SnCl_6_, Cs_2_SnBr_6_, and Cs_2_SnI_6_ compounds, specifically at 19.45 eV, 18.88 eV, and 18.31 eV, respectively. Across all configurations, it is evident that as the size of the Z-anions increases, the optical loss shifts toward lower photon energies, commonly referred to as a redshift. Among the four materials, Fig. 3(d) demonstrates that Cs_2_SnCl_6_ exhibits the greatest energy loss.

### 3.2 SCAPS-1D simulation analysis

Table 2 presents the optimized PV parameters corresponding to absorber thicknesses 1000 nm for each ETL configuration.

**Table 2:** Utilized data sources for the device structure simulation

Parameters (units)	FTO^64^	IGZO^65^	SnS_2_ (ref. 66)	CdS^67^	ZnS^68^	Cs_2_SnCl_6_	Cs_2_SnBr_6_	Cs_2_SnI_6_
Thickness (nm)	50	50	50	50	50	1000	1000	1000
Bandgap (eV)	3.6	3.050	2.24	2.40	2.80	1.652	1.358	0.228
Electron affinity (eV)	4	4.160	4.24	4.20	3.80	3.620	4.250	4.642
Dielectric permittivity	9	10	10	10	9	4.520	3.660	5.750
CB effective DOS (cm^−3^)	2.2 × 10^18^	5 × 10^18^	2.2 × 10^18^	2.8 × 10^19^	2.2 × 10^18^	1.89 × 10^17^	1.890 × 10^17^	1.848 × 10^17^
VB effective DOS (cm^−3^)	1.8 × 10^19^	5 × 10^18^	1.8 × 10^19^	2.8 × 10^19^	1.8 × 10^19^	1.629 × 10^18^	1.392 × 10^18^	1.258 × 10^18^
Electron mobility (cm^2^ V^−1^ s^−1^)	100	15	50	100	100	25	25	30
Hole mobility (cm^2^ V^−1^ s^−1^)	25	10	50	25	25	20	25	30
Donor density, ND (cm^−3^)	5 × 10^18^	1 × 10^18^	1 × 10^17^	1 × 10^18^	1 × 10^17^	0	0	0
Acceptor density, NA (cm^−3^)	0	0	0	0	0	1 × 10^17^	1 × 10^17^	1 × 10^17^
Bulk defect density, Nt (cm^−3^)	10^14^	1 × 10^15^	1 × 10^14^	10^14^	10^14^	10^12^	10^12^	10^12^

#### 3.2.1 Optimization of the thickness of the absorber layer

**Fig. 4:**
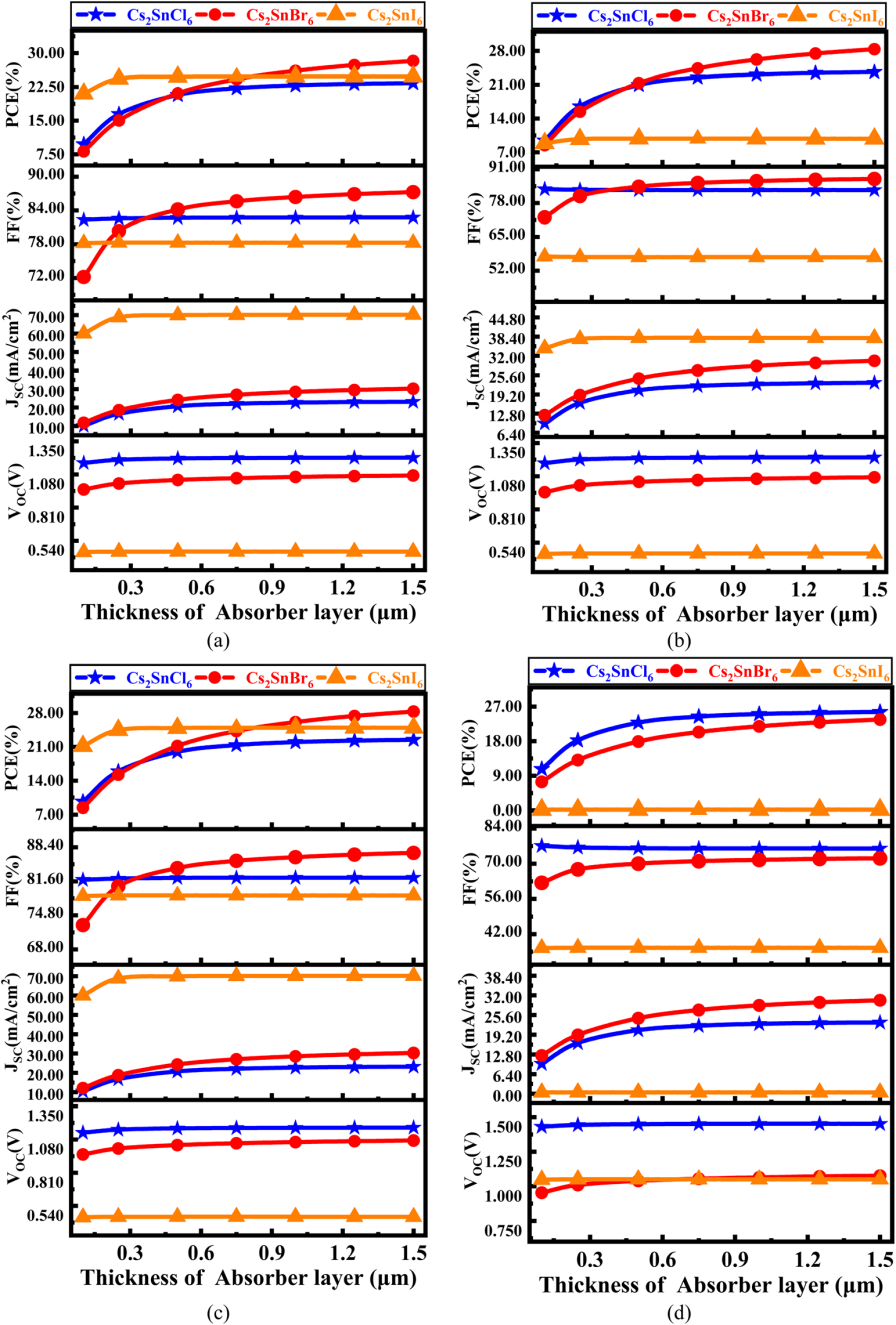
Thickness dependent PV parameters for different Cs_2_SnZ_6_ absorber layer with (a) CdS, (b) IGZO, (c) SnS_2_, and (d) ZnS ETL.

Fig. 4 comprehensively illustrates the impact of varying the absorber layer thickness from 0 μm to 1.5 μm on the performance optimization of the simulated lead-free perovskite-inspired PV devices.

In Fig. 4(a), the effect of absorber thickness on device performance using CdS as the ETL is analyzed. The variation in thickness directly alters the built-in electric field strength and modulates the charge separation and transport dynamics. The *V*_OC_ for Cs_2_SnCl_6_ increases from 1.174 V to 1.216 V, and for Cs_2_SnBr_6_, from 0.957 V to 1.070 V. These enhancements are primarily attributed to improved charge carrier extraction and reduced interfacial recombination. In contrast, Cs_2_SnI_6_ displays a relatively stable *V*_OC_, suggesting that this parameter is less sensitive to thickness-induced changes in the internal electric field for this specific absorber. The *J*_SC_ increases markedly from 11.72 to 23.18 mA cm^−2^ for Cs_2_SnCl_6_ and from 11.76 to 30.24 mA cm^−2^ for Cs_2_SnBr_6_. These improvements reflect increased photon absorption and enhanced photogenerated carrier collection. Cs_2_SnI_6_ maintains a consistently high *J*_SC_, which is characteristic of its narrow bandgap and high intrinsic absorption coefficient.

At absorber thicknesses ≥1000 nm, a saturation effect in spectral absorption is evident, accompanied by a rise in recombination losses, particularly for devices with lower mobility ETLs. This behavior aligns with previous experimental and theoretical studies. The FF exhibits a linear upward trend for Cs_2_SnCl_6_ and Cs_2_SnI_6_, indicating improved diode quality and lower series resistance. For Cs_2_SnBr_6_, the FF rises significantly from 72.17% to 87.24%, pointing to superior charge transport properties at increased thicknesses. Consequently, the PCE demonstrates a substantial improvement across all absorber types with increased thickness. Specifically, the PCE is increases from 9.76% to 23.32% for Cs_2_SnCl_6_, from 8.12% to 28.26% for Cs_2_SnBr_6_, and from 21.01% to 24.80% for Cs_2_SnI_6_, affirming the beneficial role of optimized thickness in enhancing photon management and charge collection.

Fig. 4(b) focuses on devices utilizing IGZO as the ETL. This configuration shows an increase in *V*_OC_ from 1.181 V to 1.233 V and *J*_SC_ from 9.53 mA cm^−2^ to 20.07 mA cm^−2^. The FF follows a nearly linear growth, and the PCE improves from 8.88% to 23.61%. For Cs_2_SnBr_6_, the *V*_OC_ increases from 0.949 V to 1.070 V, while the *J*_SC_ rises sharply from 12.27 mA cm^−2^ to 30.12 mA cm^−2^, attributed to the excellent carrier mobility and transparency of IGZO facilitating enhanced optical and electrical performance. The FF improves from 72.54% to 87.29%, and the PCE reaches up to 28.34%. Cs_2_SnI_6_ exhibits almost constant trend across all four parameters, emphasizing the stable interfacial energetics and minimal recombination losses with IGZO as ETL.

Fig. 4(c) explores the performance with SnS_2_ as the ETL. The *V*_OC_ for all absorbers shows an approximately linear increase with thickness, which reflects the gradual reduction in defect-assisted recombination at the interface. The *J*_SC_ increases from 10.37 mA cm^−2^ to 23.18 mA cm^−2^ for Cs_2_SnCl_6_ and from 12.06 mA cm^−2^ to 30.27 mA cm^−2^ for Cs_2_SnBr_6_, primarily due to enhanced optical absorption. Notably, Cs_2_SnI_6_ continues to exhibit a very high *J*_SC_ across all thickness values, further confirming its excellent photon harvesting capacity due to its narrow bandgap. The FF exhibits a linear trend for Cs_2_SnCl_6_ and Cs_2_SnI_6_, while for Cs_2_SnBr_6_, it increases significantly from 72.86% to 87.27%, demonstrating excellent interface passivation and charge extraction with SnS_2_. Consequently, the PCE values rise appreciably: from 9.64% to 22.44% for Cs_2_SnCl_6_, from 8.42% to 28.29% for Cs_2_SnBr_6_, and from 21.14% to 24.95% for Cs_2_SnI_6_.

Fig. 4(d) evaluates the variation in absorber thickness for devices employing ZnS as the ETL. The *V*_OC_ follows a linear trend for all three absorbers, with Cs_2_SnBr_6_ increasing from 0.931 V to 1.076 V, and Cs_2_SnCl_6_ and Cs_2_SnI_6_ remaining relatively constant around 1.08 V and 1.43 V, respectively. For Cs_2_SnCl_6_, *J*_SC_ increases from 9.74 mA cm^−2^ to 23.17 mA cm^−2^, the FF rises from 62.40% to 72.15%, and the PCE improves from 10.74% to 25.58%. Similarly, for Cs_2_SnBr_6_, *J*_SC_ increases from 12.42 mA cm^−2^ to 30.42 mA cm^−2^, but the FF slightly decreases from 77.09% to 76.02%, possibly due to charge accumulation or interfacial defects. Despite this, the PCE improves significantly from 7.39% to 23.62%.

In stark contrast, device with ZnS ETL suffer from a very low *J*_SC_ (∼0.45 mA cm^−2^), attributed to unfavorable band alignment and strong charge blocking at the ZnS/absorber interface. The FF, however, follows a linear increase starting from 36.48%, and the PCE rises gradually from 0.175%, albeit remaining considerably lower than those of other configurations.

#### 3.2.2 Optimization of doping concentration of the absorber layer

**Fig. 5:**
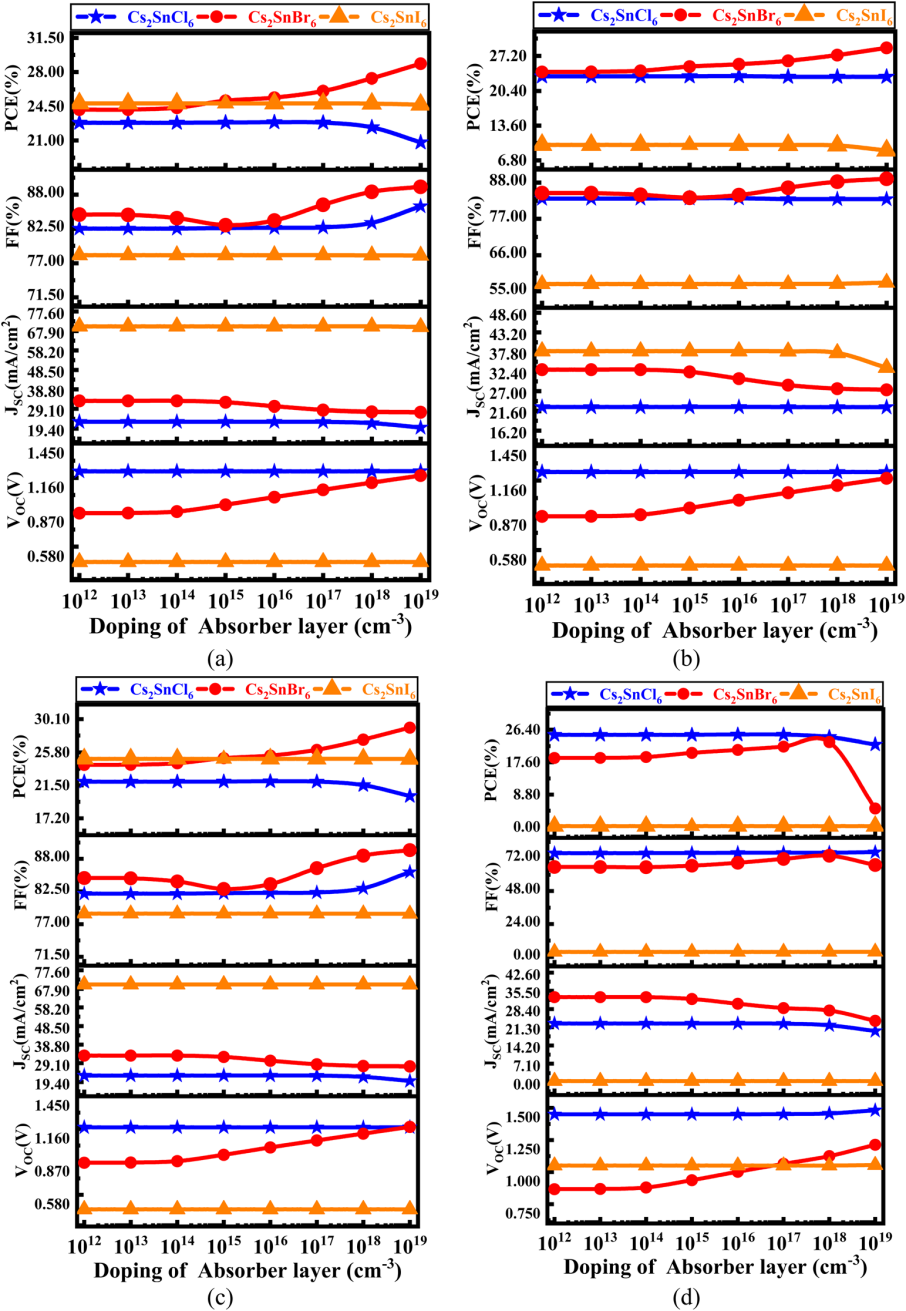
Variation in PV parameters with different doping concentrations having various Cs_2_SnZ_6_ absorber layers for (a) CdS, (b) IGZO, (c) SnS_2_, and (d) ZnS ETL.

The effect of doping concentration in Cs_2_SnCl_6_, Cs_2_SnBr_6_, Cs_2_SnI_6_ within a range of 10^12^ to 10^19^ cm^−3^ with different ETLs is shown in Fig. 5.

In Fig. 5(a), the impact of varying the doping concentration of the absorber with CdS as the ETL is shown. For Cs_2_SnCl_6_, the *V*_OC_ is 1.215 V and follows a linear trend. The *J*_SC_ decreases from 22.76 mA cm^−2^ to 19.86 mA cm^−2^, the FF increases from 82.52% to 86.12%, and the PCE decreases from 22.82% to 20.81%. For Cs_2_SnBr_6_, the *V*_OC_ increases from 0.862 V to 1.781 V, the *J*_SC_ decreases from 33.03 mA cm^−2^ to 27.62 mA cm^−2^, the FF increases from 84.78% to 89.26%, and the PCE increases from 24.16% to 28.85%. For Cs_2_SnI_6_, the *V*_OC_, *J*_SC_, FF, and PCE all show a linear trend. Fig. 5(b) shows the variation in absorber doping with IGZO as the ETL. For Cs_2_SnCl_6_ with IGZO as the ETL, the *V*_OC_ is 1.231 V and follows a linear trend. The *J*_SC_ decreases linearly, from 22.63 mA cm^−2^, the FF is 83.15% and also follows a linear trend, while the PCE is 23.19% and shows a linear variation. For Cs_2_SnBr_6_, the *V*_OC_ increases from 0.861 V to 1.178 V, the *J*_SC_ increases from 32.91 mA cm^−2^ to 27.35 mA cm^−2^, the FF increases from 84.38% to 89.18%, and the PCE increases from 24.06% to 28.76%. For Cs_2_SnI_6_, the *V*_OC_ is 0.452 V and follows a linear trend.

The *J*_SC_ decreases from 38.00 mA cm^−2^ to 33.45 mA cm^−2^, the FF is 57.21% and follows a constant trend, while the PCE decreases from 9.38% to 8.71%. Fig. 5(c) shows the variation in absorber doping with SnS_2_ as the ETL. For Cs_2_SnCl_6_, the *V*_OC_ is 1.175 V and follows a linear trend. The *J*_SC_ decreases from 22.76 mA cm^−2^ in a linear fashion, the FF increases from 82.12% to 85.75%, and the PCE decreases from 21.96% to 20.09%. For Cs_2_SnBr_6_, the *V*_OC_ increases from 0.861 V to 1.179 V, the FF rises from 84.76% to 89.46%, the *J*_SC_ decreases from 35.03 mA cm^−2^ to 27.35 mA cm^−2^, and the PCE increases from 24.16% to 28.98%. For Cs_2_SnI_6_, the *V*_OC_, *J*_SC_, FF, and PCE all follow linear trends.

Fig. 5(d) illustrates the variation in absorber doping with ZnS as the ETL. For Cs_2_SnCl_6_, the *V*_OC_ increases from 0.451 V to 1.481 V, while the *J*_SC_ decreases from 22.76 mA cm^−2^ to 19.79 mA cm^−2^. The FF starts at 75.77% and follows a linear trend, and the PCE decreases from 25.00% to 22.42%. For Cs_2_SnBr_6_, the *V*_OC_ increases from 0.872 V to 1.212 V, the FF rises from 65.60% to 73.81%, the *J*_SC_ decreases from 33.03 mA cm^−2^ to 23.78 mA cm^−2^, and the PCE decreases from 18.78% to 4.99%. For Cs_2_SnI_6_, the *V*_OC_, *J*_SC_, FF, and PCE all show constant trends.

#### 3.2.3 Influence of thickness and defect density of Cs_2_SnZ_6_ absorber

This section discusses the influence of thickness and defect density on the Cs_2_SnZ_6_ absorbers. It examines how the thickness (ranging from 0.1 to 1.5 μm) and defect density (Nt) (ranging from 10^10^ to 10^16^ cm^−3^) affect the PV output.

##### 3.2.3.1 Influence thickness and defect density of Cs_2_SnBr_6_ absorber

**Fig. 6:**
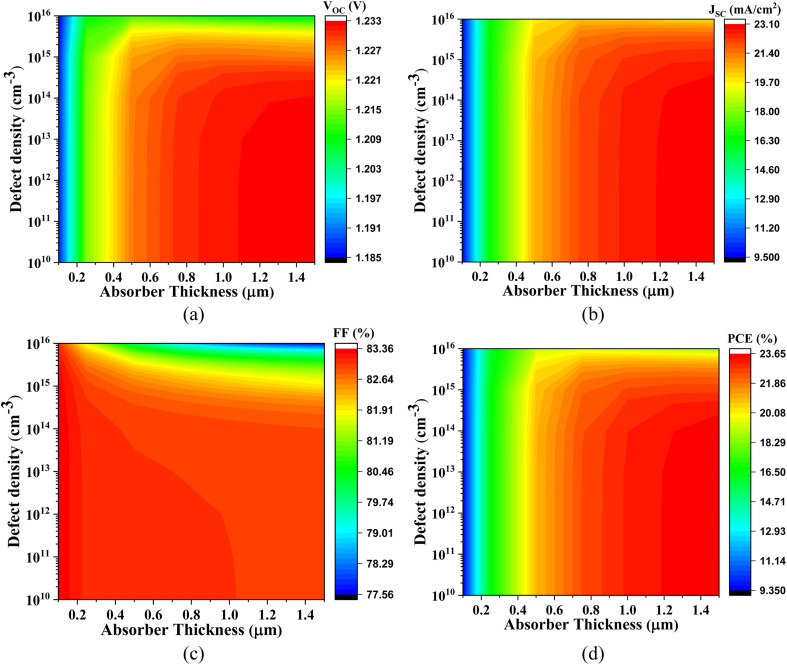
Simultaneous influence of absorber thickness and defect density on the output of Cs_2_SnCl_6_-based PSC with IGZO ETL, (a) *V*_OC_, (b) *J*_SC_, (c) FF, and (d) PCE.

The graphical representations in Fig. 6 through 9 underscore that increasing the absorber layer thickness generally enhances photovoltaic performance metrics, particularly when defect density is maintained at a low level (∼10^12^ cm^−3^). This enhancement can be attributed to two primary physical factors: improved photon absorption and more effective charge carrier generation. Thicker absorber layers increase the optical path length, allowing more incident photons to be absorbed and thus generating a higher number of electron–hole pairs. However, the benefits are contingent on the minimization of bulk and interface defects, which otherwise act as recombination centers, particularly detrimental in thicker films.

**Fig. 7:**
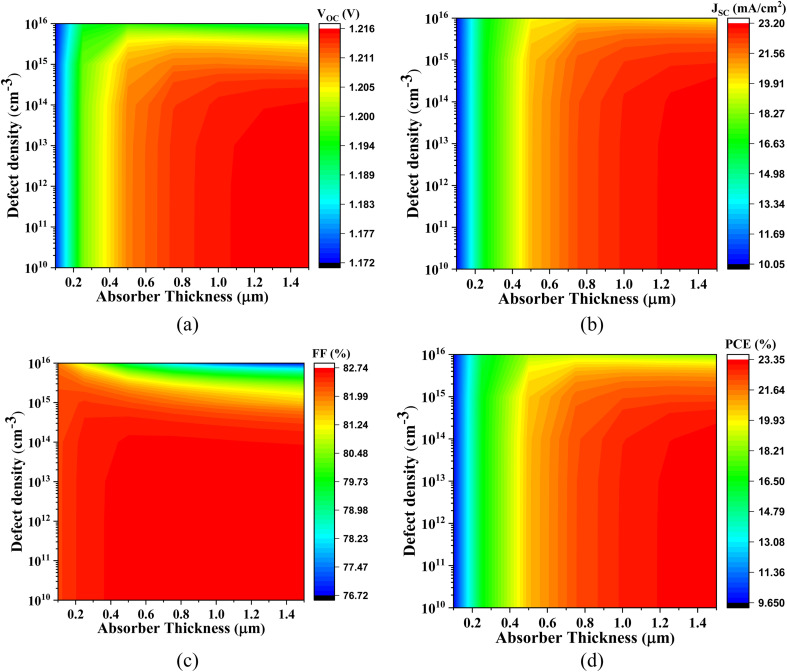
Impact of active layer thickness and defect density of Cs_2_SnCl_6_-based PSC with CdS ETL on (a) *V*_OC_, (b) *J*_SC_, (c) FF, and (d) PCE.

**Fig. 8:**
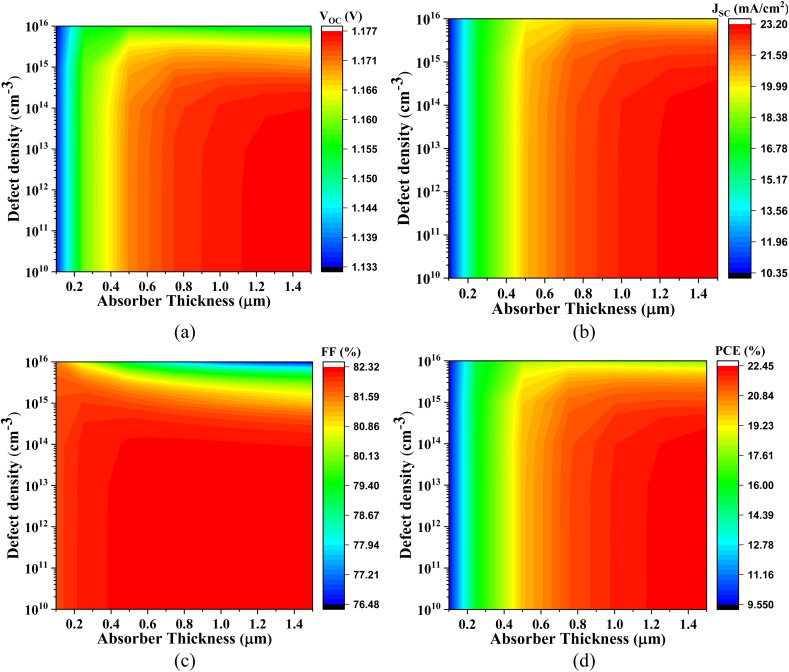
Effect of absorber layer thickness and defect density on on (a) *V*_OC_, (b) *J*_SC_, (c) FF, and (d) PCE of Cs_2_SnCl_6_-based PSC with SnS_2_ ETL.

**Fig. 9:**
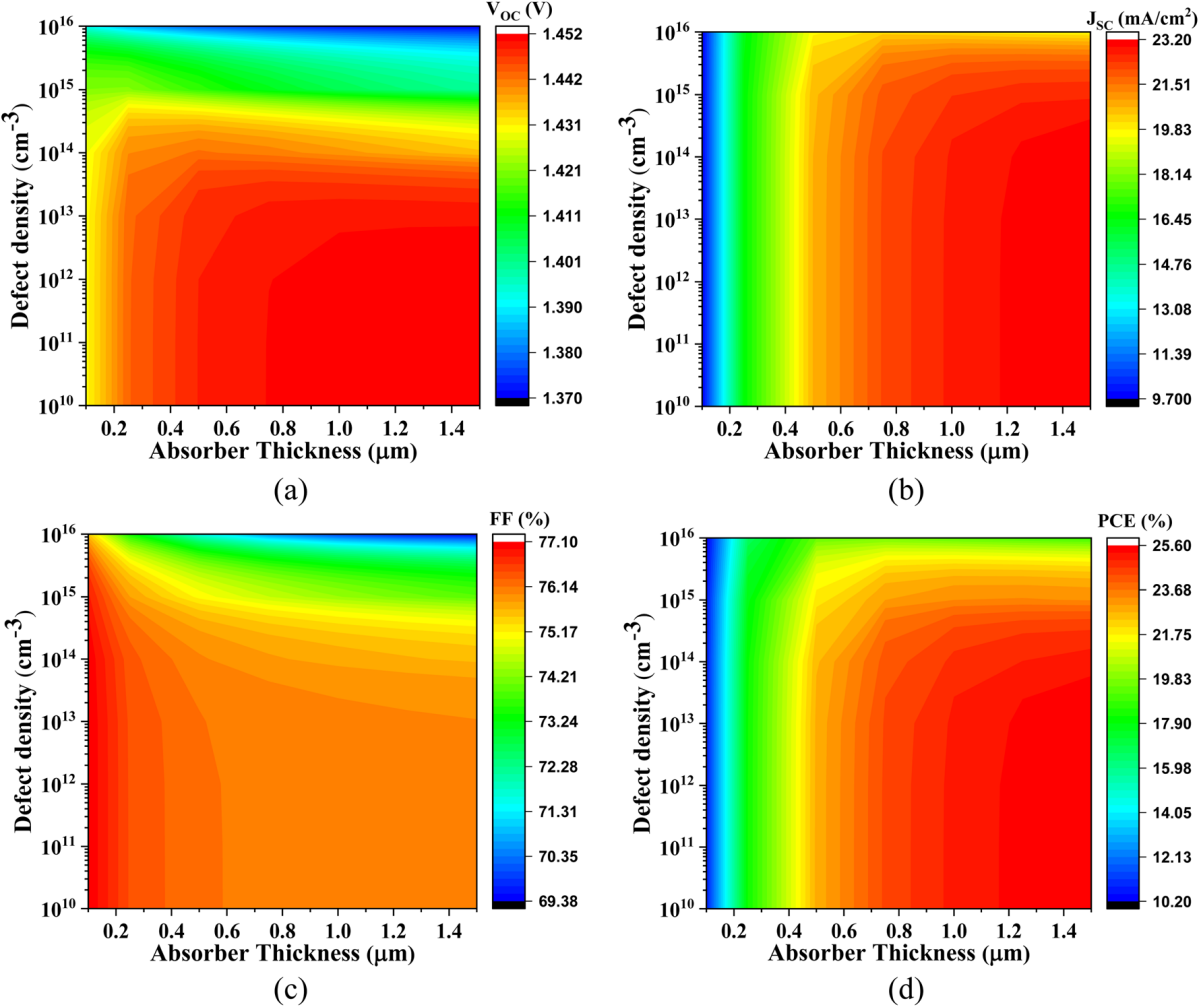
Role of the active layer thickness and defect density on (a) *V*_OC_, (b) *J*_SC_, (c) FF, and (d) PCE of Cs_2_SnCl_6_-based PSC with CdS ETL.

Fig. 6 displays the photovoltaic characteristics of Cs_2_SnCl_6_ integrated with IGZO as the ETL. With absorber thicknesses exceeding 1.0 μm and a low defect density of ∼10^12^ cm^−3^, the *V*_OC_ ranges from 1.185 V to 1.206 V, the *J*_SC_ varies from 9.741 to 23.180 mA cm^−2^, the FF spans 69.39% to 77.09%, and the PCE increases from 10.23% to 25.28%. The notable improvement in *J*_SC_ and PCE is primarily due to enhanced light absorption and reduced recombination, facilitated by the excellent band alignment and high electron mobility of IGZO, which supports efficient charge extraction.

Fig. 7 presents the performance of Cs_2_SnCl_6_-based PSC with CdS ETL. At absorber thicknesses ranging from 0.1 μm to 1.0 μm, the *V*_OC_ increases from 1.172 V to 1.216 V, *J*_SC_ ranges from 10.104 mA cm^−2^ to 23.179 mA cm^−2^, FF improves from 76.74% to 82.73%, and PCE varies from 9.76% to 23.33%. These improvements are physically justified by the favorable conduction band offset between CdS and Cs_2_SnCl_6_, which minimizes electron–hole recombination at the interface. The observed trends also indicate that even moderately thin absorbers can perform well when the interface quality is high and defect densities are low.

Fig. 8 demonstrates the photovoltaic behavior of the Cs_2_SnCl_6_-based PSC with SnS_2_ ETL. For absorber thicknesses between 0.1 μm and 1.0 μm and under low-defect conditions, *V*_OC_ varies from 1.135 V to 1.176 V, *J*_SC_ spans from 10.375 mA cm^−2^ to 23.179 mA cm^−2^, FF ranges from 76.74% to 82.32%, and PCE lies between 9.58% and 22.45%. Although SnS_2_ offers suitable band alignment, the relatively lower *V*_OC_ values compared to other configurations may be attributed to its moderate electron mobility and higher interface defect density, which can result in non-radiative recombination losses.

Fig. 9 presents the most promising results with the Cs_2_SnCl_6_-based PSC with ZnS ETL. With an absorber thickness of 0.1–1.0 μm and defect density around 10^12^ cm^−3^, the *V*_OC_ ranges from 1.370 V to 1.451 V, *J*_SC_ increases from 9.735 mA cm^−2^ to 23.180 mA cm^−2^, FF spans 69.39% to 76.09%, and PCE reaches up to 25.58%. The superior *V*_OC_ values observed in this configuration are likely due to the excellent CB alignment between ZnS and Cs_2_SnCl_6_, which creates a substantial built-in electric field that facilitates efficient charge separation and suppresses recombination.

##### 3.2.3.2 Effect thickness and defect density of Cs_2_SnBr_6_ absorber

**Fig. 10:**
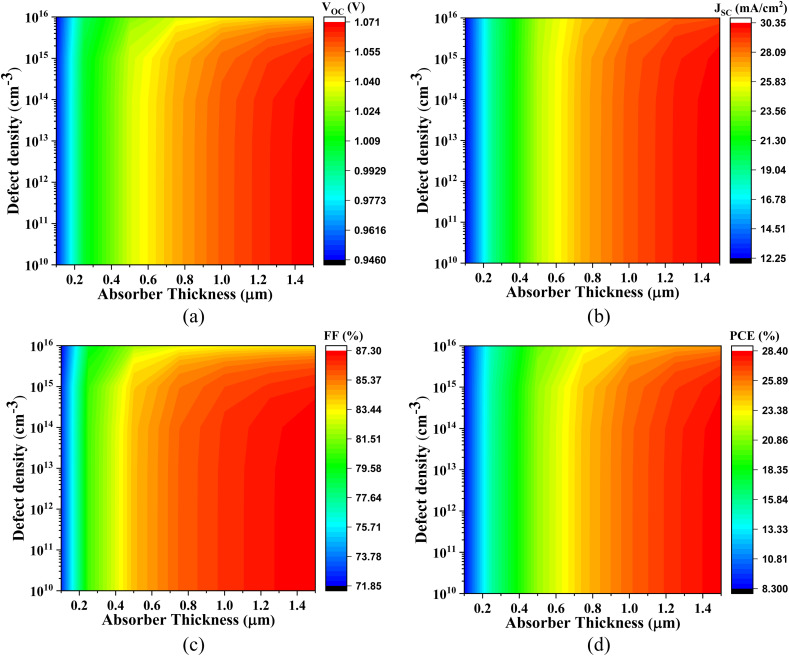
Impact of absorber thickness and defect density on (a) *V*_OC_, (b) *J*_SC_, (c) FF, and (d) PCE of Cs_2_SnBr_6_-based PSC with IGZO ETL.

Fig. 10 through 13 comprehensively illustrate the impact of defect density and absorber layer thickness on critical photovoltaic parameters for Cs_2_SnBr_6_-based solar cells employing various ETLs. Subfigures (a–d) within each figure delineate how these performance parameters evolve as a function of absorber thickness (ranging from 0.1 to 1.5 μm) and defect density, thereby enabling a detailed comparative assessment.

**Fig. 11:**
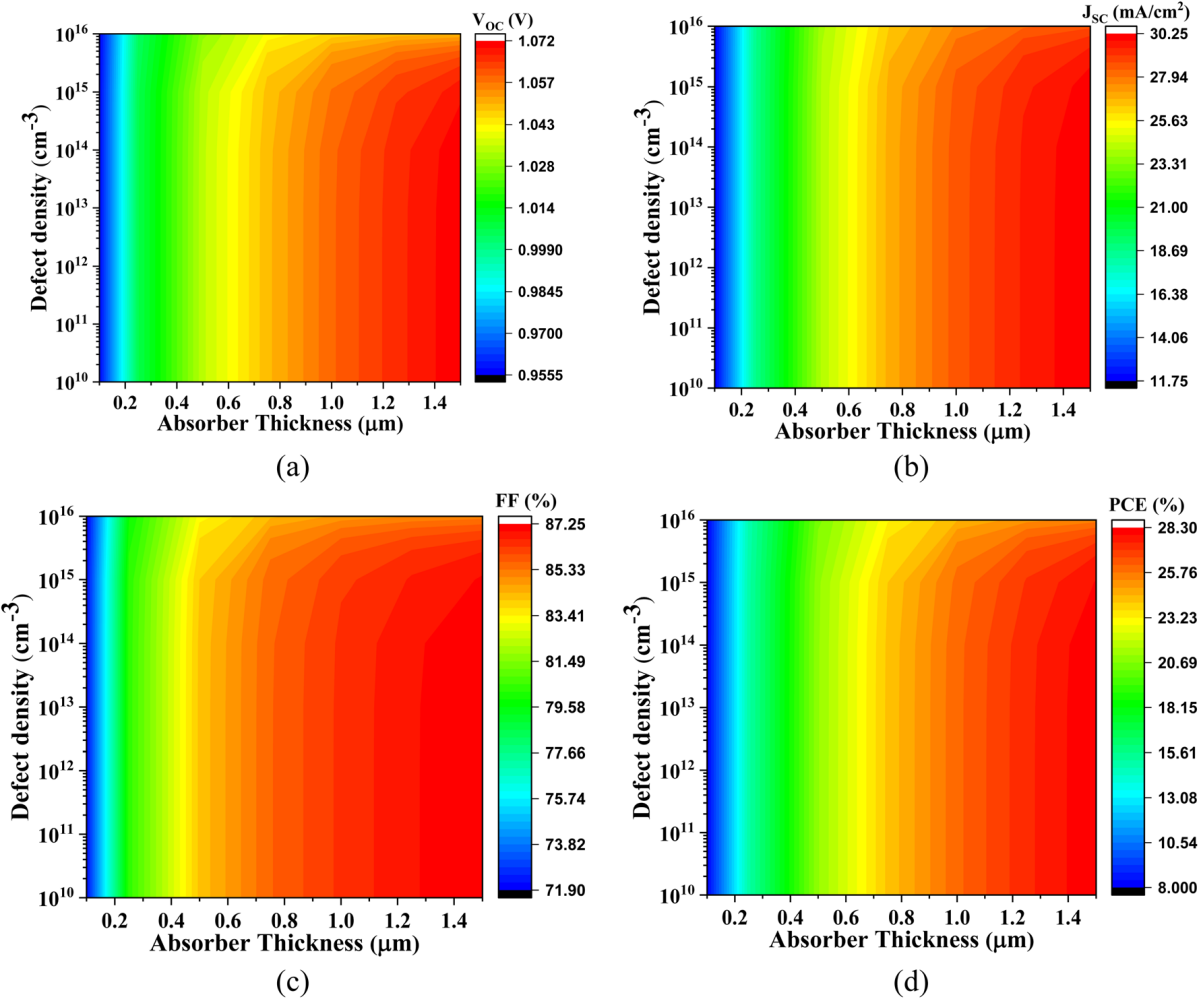
Impact of active layer thickness and defect density on (a) *V*_OC_, (b) *J*_SC_, (c) FF, and (d) PCE of Cs_2_SnBr_6_-based PSC with CdS ETL.

**Fig. 12:**
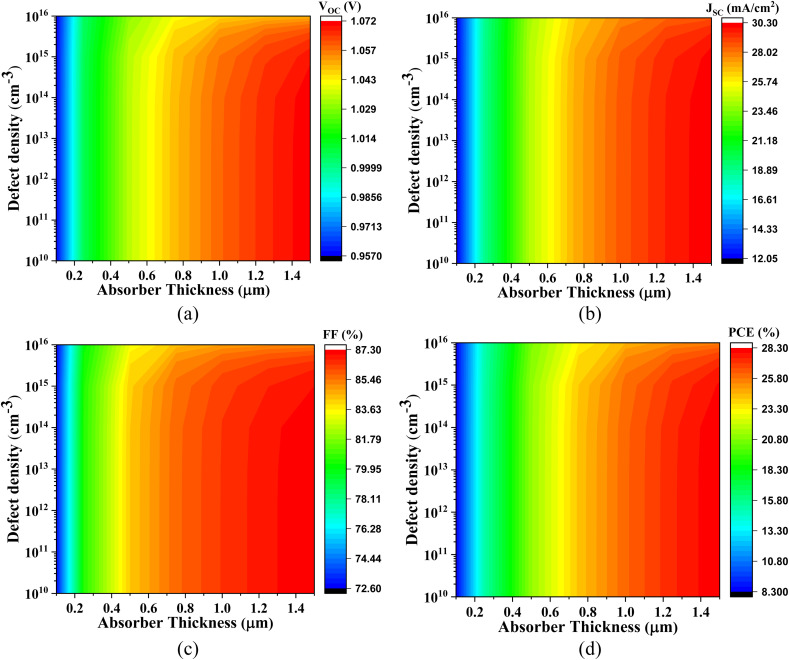
Effect of active layer thickness and defect density on (a) *V*_OC_, (b) *J*_SC_, (c) FF, and (d) PCE of Cs_2_SnBr_6_-based PSC with SnS_2_ ETL.

**Fig. 13:**
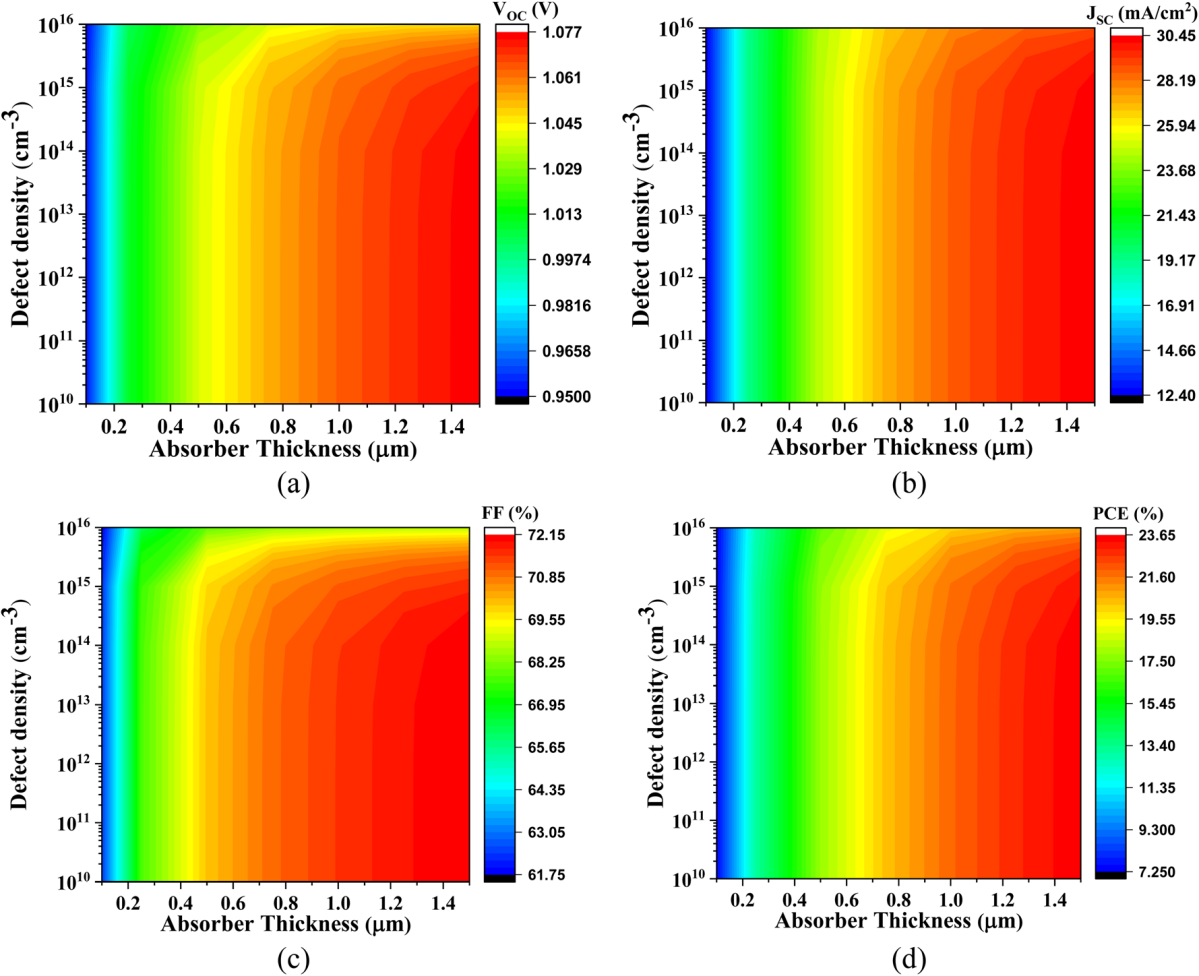
Impact of absorber thickness and defect density on (a) *V*_OC_, (b) *J*_SC_, (c) FF, and (d) PCE of Cs_2_SnBr_6_-based PSC with ZnS ETL.

Fig. 10 presents the performance behavior of the Cs_2_SnBr_6_-based PSC with IGZO ETL. The *V*_OC_ varies from 0.946 V to 1.071 V, attributable to improved internal electric field strength and reduced interfacial recombination with increasing thickness and low defect densities. The *J*_SC_ spans from 12.269 mA cm^−2^ to 30.269 mA cm^−2^, driven by enhanced photon absorption and more efficient photogenerated carrier transport in thicker absorber layers. The FF increases from 71.88% to 87.29%, primarily due to the suppression of trap-assisted recombination and improved carrier extraction dynamics. Consequently, the PCE improves significantly from 8.34% to 28.34%. However, higher defect densities introduce non-radiative recombination centers, leading to degraded charge collection efficiency and diminished values across all photovoltaic parameters.

Fig. 11 highlights the performance of the Cs_2_SnBr_6_-based PSC with CdS ETL. The *V*_OC_ increases from 0.956 V to 1.072 V, facilitated by favorable CB alignment and a strong built-in potential that minimizes carrier recombination. The *J*_SC_ increases from 11.75 mA cm^−2^ to 30.25 mA cm^−2^ with absorber thickening, due to greater light absorption and longer carrier diffusion lengths. The FF improves from 71.90% to 87.25%, reflecting reduced series resistance and enhanced interfacial charge transport. Accordingly, the PCE increases from 8.00% to 28.34%. As with other structures, the rise in defect density deteriorates all key parameters due to an escalation in bulk recombination and a decline in quasi-Fermi level splitting.

Fig. 12 demonstrates the performance metrics for the Cs_2_SnBr_6_-based PSC with SnS_2_ ETL. Here, the *V*_OC_ ranges from 0.957 V to 1.070 V, influenced by interfacial recombination dynamics and electrostatic potential alignment. The *J*_SC_ improves from 12.05 mA cm^−2^ to 30.30 mA cm^−2^, stemming from effective photogeneration and reduced carrier trapping in thicker absorbers. The FF rises from 72.55% to 87.30%, reflecting efficient charge collection at the ETL interface. These favorable characteristics translate into a notable increase in PCE from 8.30% to 28.30%. Nevertheless, performance degrades at higher defect densities, as recombination pathways become more pronounced, especially at mid-gap states.

Fig. 13 depicts the photovoltaic behavior of Cs_2_SnBr_6_-based PSC with ZnS ETL. The *V*_OC_ increases from 0.95 V to 1.08 V with increasing absorber thickness, attributed to reduced recombination losses and improved electrostatic potential profiles. The *J*_SC_ ranges from 12.43 mA cm^−2^ to 30.42 mA cm^−2^, due to enhanced optical absorption and improved carrier lifetime. However, the FF, which varies from 61.75% to 72.12%, is comparatively lower than in other configurations—likely due to unfavorable energy band alignment and increased resistive losses at the Cs_2_SnBr_6_/ZnS interface. As a result, the PCE ranges from 7.29% to 23.62%. Once again, increased defect densities lead to a consistent decline across all metrics, reaffirming the critical role of material quality in achieving high-performance lead-free perovskite photovoltaics.

##### 3.2.3.3 Influence thickness and defect density of Cs_2_SnI_6_ absorber

**Fig. 14:**
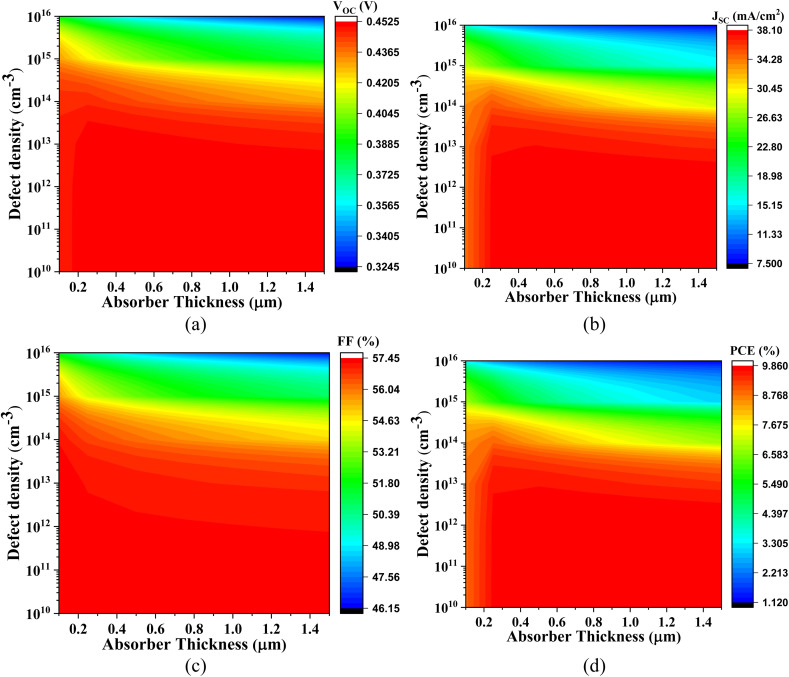
Impact of active layer thickness and defect density on (a) *V*_OC_, (b) *J*_SC_, (c) FF, and (d) PCE of Cs_2_SnI_6_-based PSC with IGZO ETL.

**Fig. 15:**
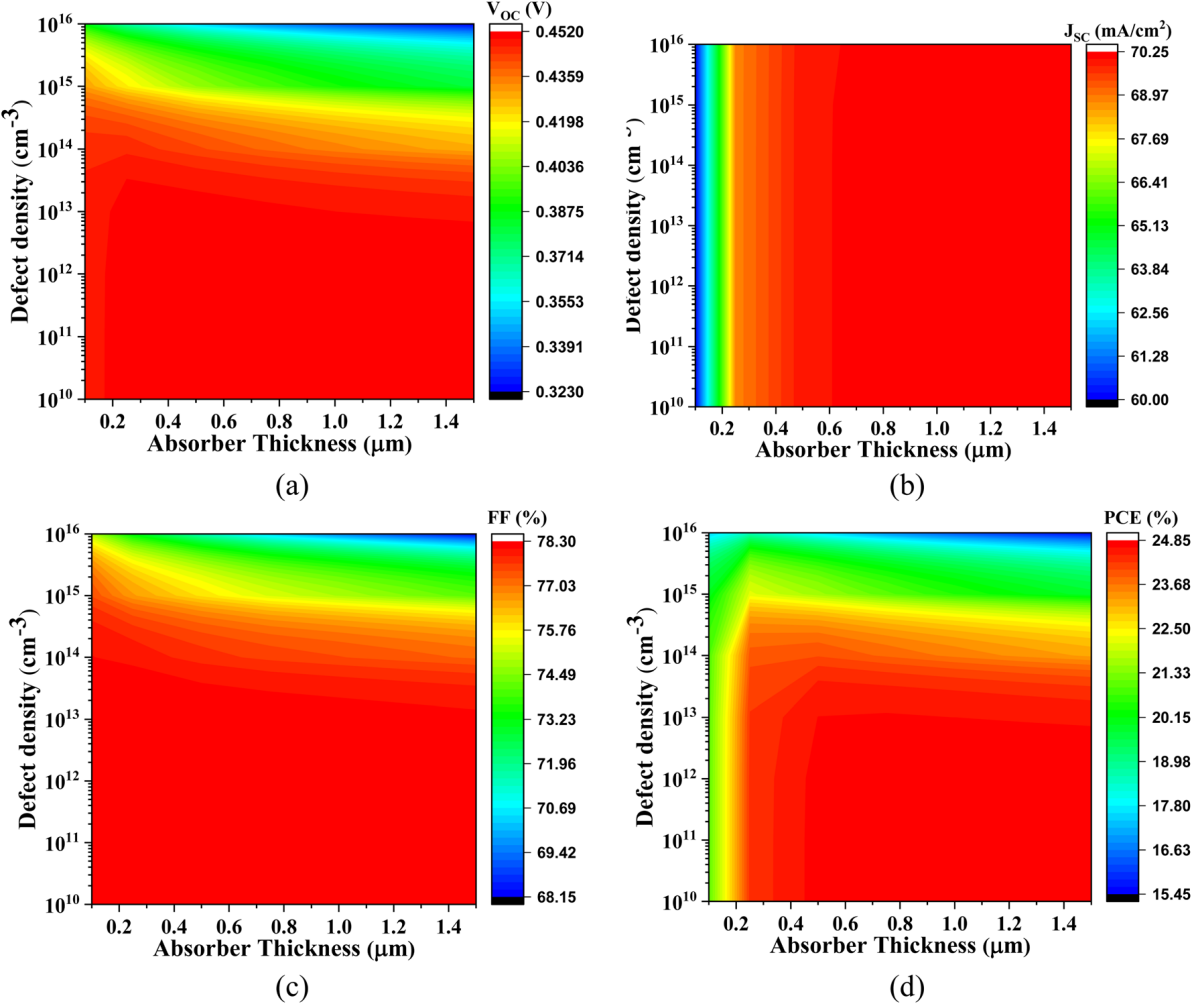
Influence of absorber thickness and defect density on (a) *V*_OC_, (b) *J*_SC_, (c) FF, and (d) PCE of Cs_2_SnI_6_-based PSC with CdS ETL.

**Fig. 16:**
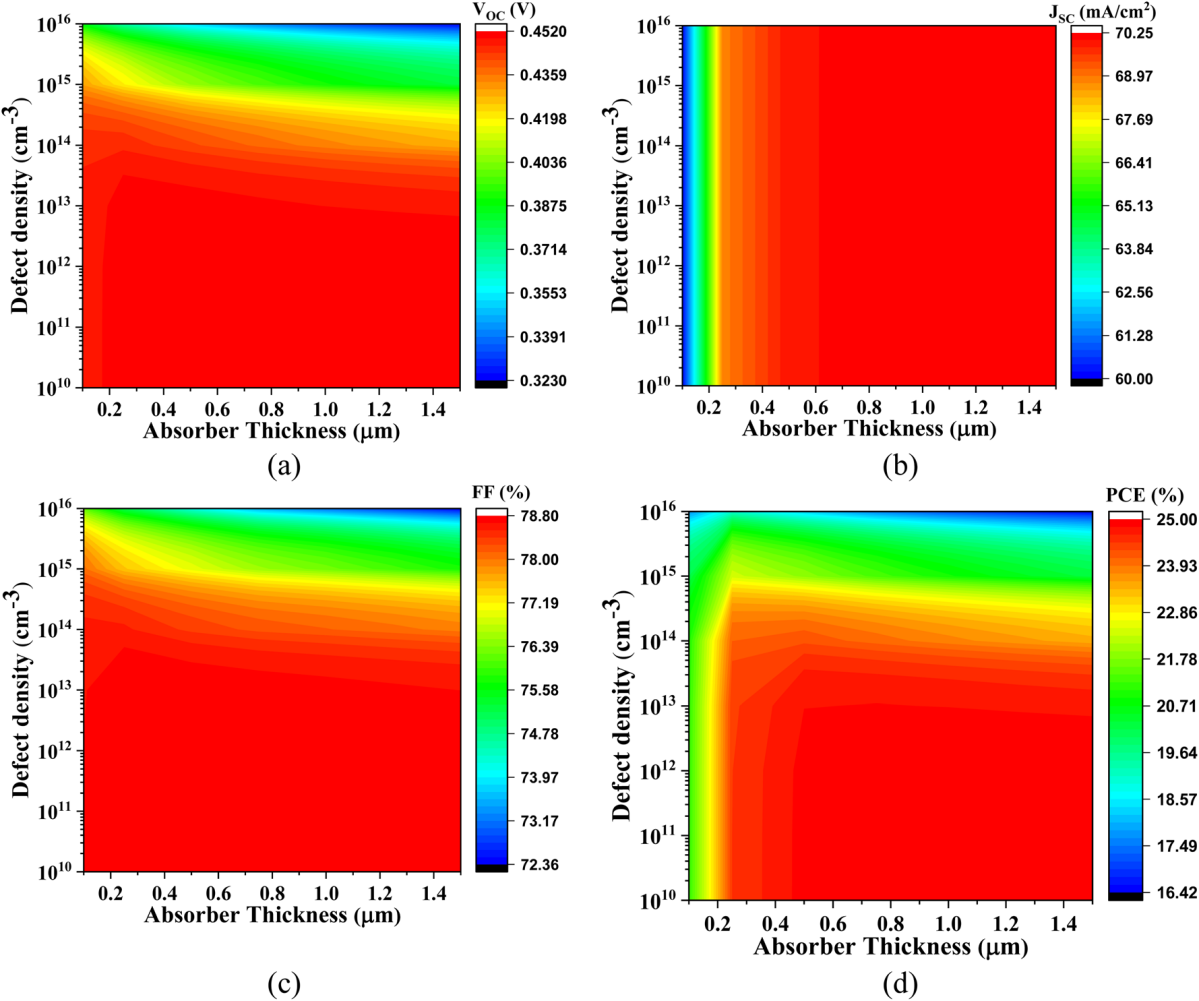
Impact of absorber layer thickness and defect density on (a) *V*_OC_, (b) *J*_SC_, (c) FF, and (d) PCE of Cs_2_SnI_6_-based PSC with SnS_2_ ETL.

**Fig. 17:**
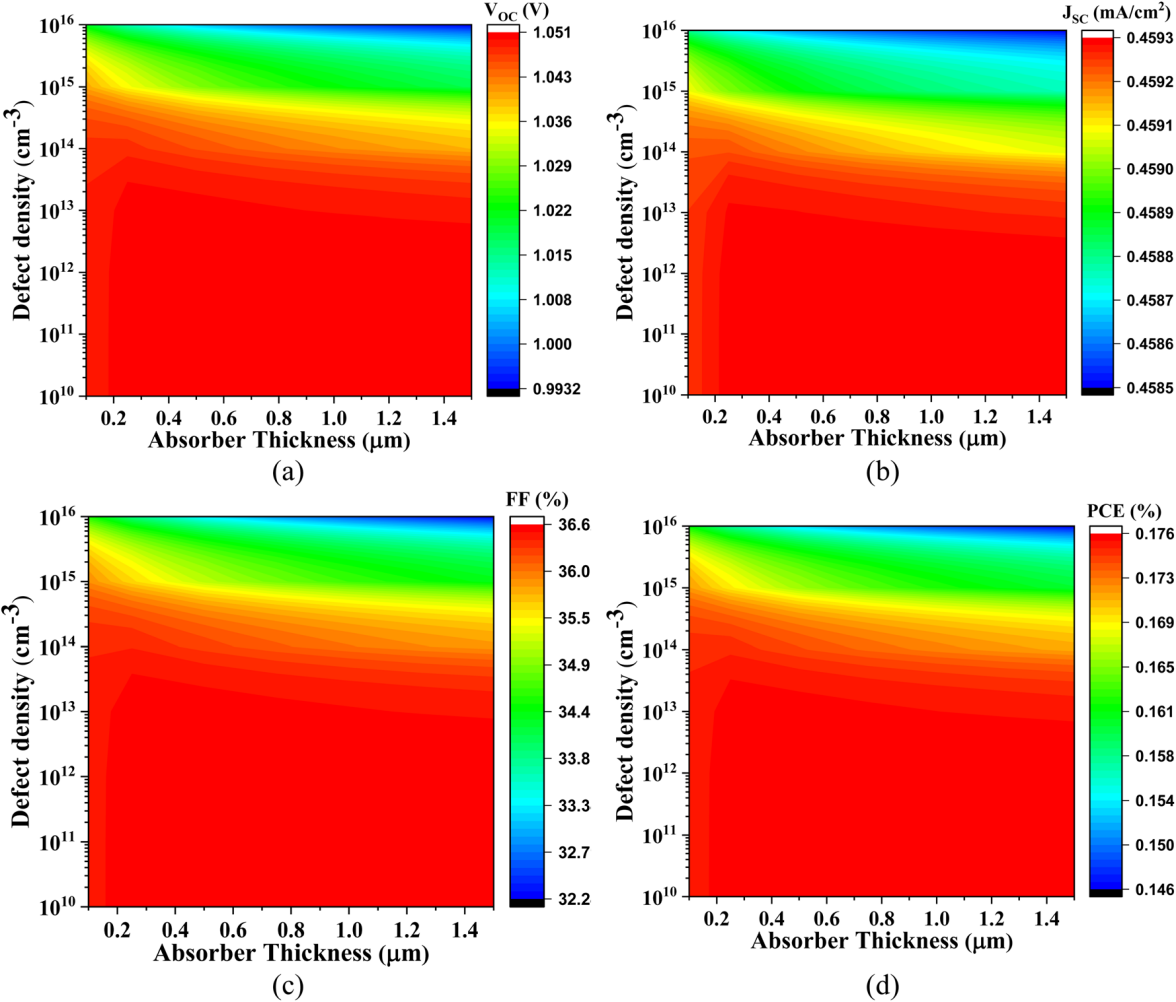
Effect of active layer thickness and defect density on (a) *V*_OC_, (b) *J*_SC_, (c) FF, and (d) PCE of Cs_2_SnI_6_-based PSC with ZnS ETL.

Owing to the intrinsically narrow bandgap of the Cs_2_SnI_6_ absorber, the PV performance characteristics of this material interfaced with various ETLs are systematically presented in Fig. 14–17, respectively.

Fig. 14 illustrates the device behavior of the Cs_2_SnI_6_-based PSC with IGZO ETL. The *V*_OC_ ranges from 0.32 V to 0.45 V, constrained primarily by the low quasi-Fermi level splitting associated with the narrow bandgap of Cs_2_SnI_6_. In contrast, the short-circuit current density *J*_SC_ spans a remarkably high range, from 60.04 mA cm^−2^ to 70.20 mA cm^−2^, driven by the strong photon absorption capacity in the visible to near-infrared spectral regions. The FF ranges from 76.17% to 78.78%, reflecting moderate carrier transport efficiency and minimal recombination losses. As a result, the PCE improves from 16.42% to 25.98%, highlighting the absorber's superior light-harvesting ability despite its limited photovoltage.

Table 3 presents the optimized PV performance parameters for Cs_2_SnCl_6_-, Cs_2_SnBr_6_-, and Cs_2_SnI_6_-based devices employing different ETLs. For Cs_2_SnCl_6_-based PSC, ZnS yields the highest PCE of 25.05%, attributed to its relatively high *V*_OC_ = 1.450 V, despite a modest FF (76.05%). Cs_2_SnBr_6_-based devices achieve comparable maximum efficiencies, with IGZO delivering the highest PCE of 26.22%, driven by its superior *J*_SC_ (28.65 mA cm^−2^) and FF (86.44%), although the *V*_OC_ remains modest. In contrast, Cs_2_SnI_6_ exhibits the most significant variation among ETLs: while SnS_2_ and CdS facilitate excellent performance (PCEs ≈ 25%) due to very high *J*_SC_ values (>70 mA cm^−2^), the ZnS-based device performs poorly (PCE = 0.18%) owing to a drastic reduction in *J*_SC_ and FF, indicating strong charge blocking and poor band alignment.

**Table 3:** Obtained PV output with optimized parameters

ETL	Parameters			
	*V*_OC_ (V)	*J*_SC_ (mA cm^−2^)	FF (%)	PCE (%)
**Cs**_**2**_ **SnCl**_**6**_ **-based PSC**				
IGZO	1.232	22.60	83.00	23.11
SnS_2_	1.175	22.71	82.30	21.97
CdS	1.215	22.71	82.72	22.83
ZnS	1.450	22.71	76.05	25.05

**Cs**_**2**_ **SnBr**_**6**_ **-based PSC**				
IGZO	1.059	28.65	86.44	26.22
SnS_2_	1.059	25.54	86.41	26.12
CdS	1.059	28.50	86.37	26.07
ZnS	1.063	28.74	71.52	21.87

**Cs**_**2**_ **SnI**_**6**_ **-based PSC**				
IGZO	0.452	37.99	57.22	9.82
SnS_2_	0.451	70.17	78.78	24.95
CdS	0.451	70.18	78.28	24.80
ZnS	1.050	0.46	36.60	0.18

Fig. 15 depicts the PV behavior of Cs_2_SnI_6_-based PSC with CdS ETL, where the *V*_OC_ similarly varies between 0.32 V and 0.45 V, again limited by the narrow absorber bandgap. The *J*_SC_ increases from 60.00 mA cm^−2^ to 70.25 mA cm^−2^, benefiting from the extended spectral absorption. However, the FF ranges from 68.15% to 78.30%, slightly lower than that of IGZO, possibly due to increased interface recombination or less favorable band alignment at the CdS/Cs_2_SnI_6_ interface. Accordingly, the PCE ranges from 15.45% to 24.85%.

Fig. 16 presents the photovoltaic response of Cs_2_SnI_6_-based PSC with SnS_2_ ETL. The *V*_OC_ values range from 0.323 V to 0.452 V, following similar trends dictated by the fundamental bandgap limitations. The *J*_SC_ values remain robust, ranging from 60.00 mA cm^−2^ to 70.20 mA cm^−2^, due to effective light absorption and favorable optical constants. The FF spans from 72.40% to 78.80%, indicating improved charge extraction and reduced series resistance relative to CdS. These features collectively yield a PCE between 16.40% and 25.00%, signifying a promising device configuration.

Fig. 17, in contrast, delineates the device metrics for the Cs_2_SnI_6_-based PSC with ZnS ETL. In this configuration, the *V*_OC_ shows unusually high values, ranging from 0.99 V to 1.05 V, which may arise due to an unfavorable energy band offset at the ZnS/Cs_2_SnI_6_ interface that hinders effective carrier recombination and artificially elevates the *V*_OC_. However, this comes at a significant trade-off: the *J*_SC_ is extremely low, varying only between 0.45 mA cm^−2^ and 0.46 mA cm^−2^, which is attributed to excessive interfacial carrier blocking and poor electron injection efficiency, owing to the large bandgap and poor energy level alignment of ZnS. The FF ranges from only 32.24% to 36.61%, likely due to high series resistance and severe charge accumulation at the interface. Consequently, the PCE drops sharply to a mere 0.16–0.18%, indicating the unsuitability of ZnS as an ETL for Cs_2_SnI_6_-based solar cells.

### 3.3 *J*–*V* and QE characteristics corresponding to variations in absorber layer thickness of the optimized device

**Fig. 18:**
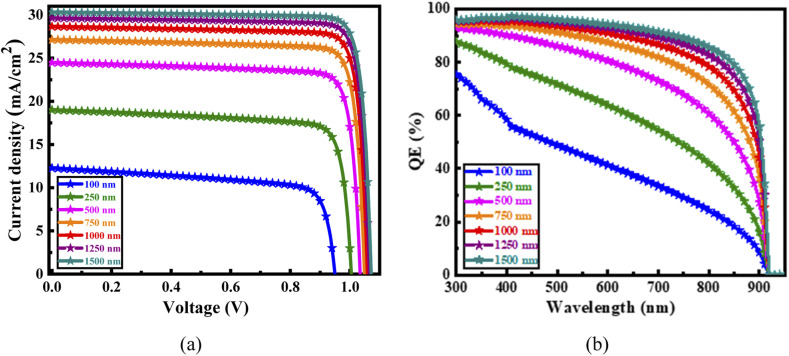
(a) Current–voltage and (b) quantum efficiency characteristics of the optimized device as a function of absorber layer thickness.

The *J*–*V* and QE characteristics presented in Fig. 18(a) and (b), respectively, illustrate the influence of varying the Cs_2_SnBr_6_ absorber layer thickness on the photovoltaic performance of solar cells employing an IGZO ETL. As shown in Fig. 18(a), both the *J*_SC_ and *V*_OC_ increase progressively with absorber thickness from 100 nm to 1500 nm, primarily due to enhanced light absorption and reduced interface recombination. The device with a 1500 nm thick absorber achieves the highest *J*_SC_ and *V*_OC_, reflecting superior carrier generation and collection.^69^ However, the performance improvement beyond 1000 nm is marginal, while the potential for increased recombination and material usage becomes more significant. Therefore, 1000 nm is considered the optimal thickness, offering a favorable balance between high efficiency and material economy. The trend is further corroborated by the QE spectra in Fig. 18(b), where QE improves with thickness due to stronger photon absorption, with the 1000 nm to 1500 nm devices exhibiting the most effective spectral response across the visible and near-infrared regions. In contrast, thinner devices, particularly at 100 nm and 250 nm, exhibit noticeably lower QE and *J*_SC_ values due to insufficient absorption depth and weaker charge carrier collection.

Table 4 provides a comprehensive comparative analysis of Cs_2_SnX_6_-based perovskite solar cells, highlighting key findings from both experimental investigations and simulation-based studies to evaluate their performance and potential for photovoltaic applications.

**Table 4:** Comparative performance metrics of Cs_2_SnX_6_-based solar cell configurations^a^

Type	Structure	*V*_OC_ (V)	*J*_SC_ (mA cm^−2^)	FF (%)	PCE (%)	Ref.
E	FTO/TiO_2_/Cs_2_SnI_6_/P3HT	0.52	3.2	51.5	0.86	70
E	FTO/TiO_2_/Cs_2_SnI_6_/Dye	0.67	16.3	61.8	6.8	71
E	FTO/TiO_2_/Cs_2_SnI_6_	0.67	12.56	0.50	4.23	72
S	ITO/CuI/Cs_2_SnI_6_/PCBM	0.86	23.91	71.08	14.65	73
S	FTO/TiO_2_/Cs_2_SnI_6_/Cu_2_O	0.74	30.00	80.50	17.77	74
S	FTO/SnS_2_/Cs_2_SnI_6_	0.451	70.17	78.78	24.95	This work(optimized)
S	FTO/ZnS/Cs_2_SnCl_6_	1.45	22.71	76.05	25.05	This work(optimized)
S	FTO/IGZO/Cs_2_SnBr_6_	1.059	28.65	86.44	26.22	This work(optimized)

a E = experimental, S = simulation.

## 4. Conclusions

In this work, we conducted an in-depth first-principles and numerical evaluation of Cs_2_SnZ_6_ compounds as lead-free photovoltaic absorbers. DFT-based analyses confirmed their structural integrity, direct bandgaps, and strong optical absorption, with clear trends linking halide substitution to bandgap narrowing and enhanced light-harvesting capacity. SCAPS-1D simulations further elucidated the influence of absorber thickness, doping concentration, and defect density on device performance, revealing that absorber layers exceeding 1000 nm and low bulk defect densities significantly enhance photovoltaic efficiency. Among the investigated configurations, the Al/FTO/IGZO/Cs_2_SnBr_6_/Au combination yielded the best performance (PCE = 26.22%), balancing favorable band alignment, carrier mobility, and optical properties. Conversely, Cs_2_SnI_6_, while offering high *J*_SC_ due to its narrow bandgap, was limited by poor ETL compatibility in certain structures (*e.g.*, ZnS). Overall, this study underscores the viability of Cs_2_SnZ_6_ perovskites in eco-friendly solar technologies and provides strategic guidance for material and device-level optimization to propel the development of high-performance, lead-free perovskite solar cells.

## Conflicts of interest

There are no conflicts of interest to declare.

## Data Availability

Data will be available on request.
